# Daily temperature cycles promote alternative splicing of RNAs encoding SR45a, a splicing regulator in maize

**DOI:** 10.1093/plphys/kiab110

**Published:** 2021-03-10

**Authors:** Zhaoxia Li, Jie Tang, Diane C Bassham, Stephen H. Howell

**Affiliations:** 1 Plant Sciences Institute, Iowa State University, Ames, Iowa 50011, USA; 2 Genetics, Development and Cell Biology Department, Iowa State University, Ames, Iowa 50011, USA

## Abstract

Elevated temperatures enhance alternative RNA splicing in maize (*Zea mays*) with the potential to expand the repertoire of plant responses to heat stress. Alternative RNA splicing generates multiple RNA isoforms for many maize genes, and here we observed changes in the pattern of RNA isoforms with temperature changes. Increases in maximum daily temperature elevated the frequency of the major modes of alternative splices (AS), in particular retained introns and skipped exons. The genes most frequently targeted by increased AS with temperature encode factors involved in RNA processing and plant development. Genes encoding regulators of alternative RNA splicing were themselves among the principal AS targets in maize. Under controlled environmental conditions, daily changes in temperature comparable to field conditions altered the abundance of different RNA isoforms, including the RNAs encoding the splicing regulator SR45a, a member of the *SR45* gene family. We established an “in protoplast” RNA splicing assay to show that during the afternoon on simulated hot summer days, SR45a RNA isoforms were produced with the potential to encode proteins efficient in splicing model substrates. With the RNA splicing assay, we also defined the exonic splicing enhancers that the splicing-efficient SR45a forms utilize to aid in the splicing of model substrates. Hence, with rising temperatures on hot summer days, SR45a RNA isoforms in maize are produced with the capability to encode proteins with greater RNA splicing potential.

## Teaser

RNA splicing patterns for *SR45a*, a major RNA splicing regulator in maize, change in response to maximum daily temperature and vary during the day in response to daily temperature cycles.

## Introduction

As sessile organisms, plants face changing environmental conditions for which they have evolved mechanisms to enhance gene expression plasticity and proteome diversity. One mechanism that contributes to these adaptive properties is alternative splicing (AS). AS produces different RNA isoforms thereby enhancing the complexity of genic output and possibly the repertoire of responses to different environmental conditions (Laloum et al., 2018; Chaudhary et al., 2019; Punzo et al., 2020). AS exerts influence over all aspects of plant growth and development (de Francisco Amorim et al., 2018; Kanno et al., 2018; Szakonyi and Duque, 2018; Petrillo et al., 2020), operation of the circadian clock (James et al., 2012; Cui et al., 2014; Filichkin et al., 2014; Kwon et al., 2014; Nolte and Staiger, 2015; Marshall et al., 2016), flowering transition (Macknight et al., 2002; Cui et al., 2017; Verhage et al., 2017; Park et al., 2019; Wang et al., 2019), the light regulation of rubisco activase (Werneke et al., 1989; Yin et al., 2014), and stress responses (Ding et al., 2014; Feng et al., 2015; Thatcher et al., 2016; Jiang et al., 2017; Keller et al., 2017; Zhu et al., 2017; Laloum et al., 2018).

RNA splicing is catalyzed by spliceosomes, large complexes composed of five small RNAs and numerous proteins (Will and Luhrmann, 2011). The spliceosome is a dynamic structure and assembled on each pre-mRNA that undergoes splicing. RNA splicing is intimately associated with transcription, and cotranscriptional splicing has been found to be widespread in Arabidopsis (*Arabidopsis thaliana*; Li et al., 2020; Zhu et al., 2020). Splicing is a two-step process and in the first step the branch site adenosine attacks the 5′ splice site resulting in a cleaved 5′ exon and an intron-3′ exon lariat intermediate. In the second step, the intron is excised, and the 5′ and 3′ exons are ligated together (Warkocki et al., 2009). AS involves decisions about which introns are to be removed and which exons are to be included in the mature RNA. These decisions in plants are influenced by a group of serine/arginine-rich proteins (SR proteins) acting in *trans* (Reddy and Shad Ali, 2011) and by *cis* regulatory elements on the RNA, such as exon splicing enhancers (ESEs; Chen and Manley, 2009). SR proteins bind to ESEs through RNA recognition motifs (RRMs; Zahler et al., 1992; Graveley, 2000; Maniatis and Tasic, 2002). Twenty-one SR protein genes have been reported for maize (Rauch et al., 2014), and the maize SR proteins, in general, have one or two RRMs near their N-termini and a RS domain near their C-termini (Barta et al., 2010; Manley and Krainer, 2010; Reddy and Shad Ali, 2011). RS domains support protein-protein interactions and are intrinsically disordered regions allowing for broad binding specificity (Haynes and Iakoucheva, 2006). In mammalian cells, SR proteins have been shown to play important roles in “exon definition” splicing site selection by recruiting U1 small nuclear ribonucleoprotein (U1 snRNP) to the 5′ splice site and the U2 auxiliary factor (U2AF) complex and U2 small nuclear ribonucleoprotein (U2 snRNP) to the 3′ splice site (Chen and Manley, 2009).

The genes encoding SR proteins are themselves AS targets. Because of AS, 15 members of the SR protein gene family in Arabidopsis produce 95 different RNA isoforms, potentially increasing the complexity of this family six-fold (Palusa et al., 2007). However, not all isoforms are expected to be functional—many would likely have frame shifts leading to premature translation termination. About 1/3 of the alternatively spliced SR protein RNAs do not even make it to polysomes for translation, and may be retained in the nucleus (Palusa and Reddy, 2015).

AS events arise from five major modes of splicing: exon skipping (ES), intron retention (IR), mutually exclusive exons (MXE, in which the precursor-mRNA (pre-mRNA) contains two adjacent exons but the mature transcript contains only one or the other of the two exons), alternative 5′ donor sites (A5SS) or alternative 3′ acceptor sites (A3SS). The most frequent AS mode in plants is IR, however that depends on the tissues sampled and growth conditions of the plants (Filichkin et al., 2010; Kalyna et al., 2012; Marquez et al., 2012; Mandadi and Scholthof, 2015). More than any other mode, IR tends to produce transcripts with premature termination codons (PTCs) that may be subject to nonsense mediated decay (NMD) or that may be translated to give rise to truncated proteins (Filichkin and Mockler, 2012; Kalyna et al., 2012; Drechsel et al., 2013; Filichkin et al., 2014). Different environmental conditions can lead to changes in the frequency or patterns of AS. Palusa et al. (2007) found that heat and cold affect AS for most SR genes in Arabidopsis. They suggested that environmental stress may alter the phosphorylation status of SR proteins. It has been shown that LAMMER-type protein kinases, such as CDC-like protein kinases, phosphorylate SR proteins in vitro and overexpression of a tobacco (*Nicotiana tabacum*) LAMMER protein kinase in Arabidopsis altered the splicing pattern of some SR proteins (Palusa et al., 2007). CDC-like protein kinases in mammals have been shown to be temperature sensitive and lower body temperatures increase phosphorylation of SR proteins by these kinases both in vitro and in vivo (Haltenhof et al., 2020). Hence, the effects of heat on AS may be related to the phosphorylation state and/or nuclear localization of SR proteins.

We examined AS in maize to determine the genome-wide and gene-specific effects of elevated temperatures on AS. Plants were subjected to elevated temperatures under controlled conditions in the Enviratron (Bao et al., 2019) to simulate field conditions to which plants might be exposed on a hot summer day. We found that increasing daily temperatures had global effects of increasing the frequency of AS, in particular IRs and ESs. The genes most frequently subject to AS at elevated daily temperature were involved in RNA processing, plant development and protein modification/chromosome segregation. It was of interest to find that some of the genes most impacted by AS at elevated daily temperature encode RNA splicing regulators that themselves are involved in AS. To assess environmental effects on AS, we focused our attention on one of the splicing regulators, SR45a, a member of the serine/arginine-rich (SR) protein family, which have been used as models for the study of AS in plants (Ali et al., 2003; Ali et al., 2007; Tanabe et al., 2009; Zhang and Mount, 2009; Yoshimura et al., 2011; Mori et al., 2012; Filichkin et al., 2014; Xing et al., 2015; Jiang et al., 2017). Interestingly, we found in maize that the pattern of different RNA isoforms changed throughout the simulated day with nonproductive RNA isoforms produced in the virtual morning and potentially productive RNA isoforms produced in the warm virtual afternoon. Of special interest, we developed an “in protoplast” RNA splicing system and in using it, we found that the different SR45a RNA isoforms encoded proteins with different efficiencies in the splicing of model RNA substrates. In general, SR45a RNA isoforms with the potential to encode splicing factors with greater splicing efficiencies were produced later in the day as temperatures rose.

## Results

### The effect of elevated temperature on AS

**Figure 1:**
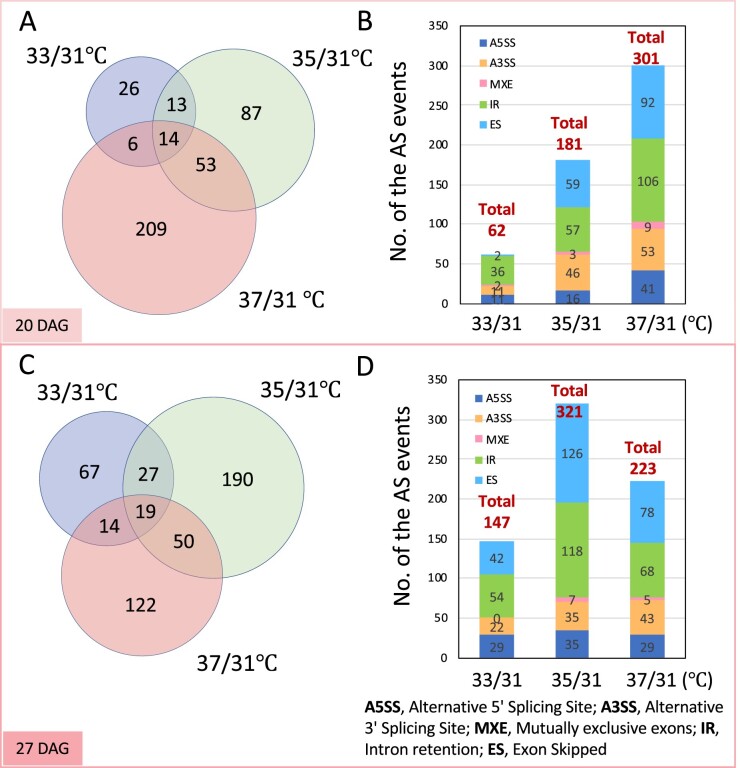
AS changes in response to increased MDTs. A and B, AS changes at developmental stage V4 (20 DAG). The proportional Venn diagram in A shows changes in abundance of DASs referring to genes affected at the different MDTs compared to 31°C MDT. B, The stacked bar graph displays changes in the types of ASs with increasing MDT. C and D, AS changes at developmental stage V5 (27 DAG). As above, the proportional Venn diagram in C shows changes in abundance of DASs referring to genes affected at the different MDTs compared to 31°C MDT, and the stacked bar graph in D displays changes in the types of DASs with increasing MDT. The threshold of |Δψ| > 5% and FDR ≤1% were used to judge DASs.

To investigate gene expression events in maize leaves in response to elevated temperature, we analyzed the frequency and pattern of AS in plants subjected to daily temperature cycles in the controlled conditions of the Enviratron (Bao et al., 2019). Following germination, maize plants were grown continuously under conditions in which temperatures were ramped up over 6 h in the virtual morning to a maximum daily temperature (MDT) of 31°C, 33°C, 35°C, or 37°C, and ramped down over 8 h in the virtual evening to 10°C below the MDT (Supplemental Figure S1). As the temperature reached MDT, RNA was extracted and analyzed for AS from the first fully expanded leaf of plants at developmental stage V4 and V5. (Developmental stages are defined by the leaf numbers visible in the leaf collars, the light-colored band located at the base of an exposed leaf blade, where the leaf blade attaches to the plant stem. For example, at the V4 stage leaf 4 is visible in the collar.) For the leaves at developmental stage V4 (20 DAG), ∼9,000 different AS events were identified in each sample, out of the ∼4,700 genes experiencing AS at that stage (Supplemental Tables S1 and S2). For the leaves at developmental stage V5 (27 DAG), ∼11,000 AS events were identified in each sample, out of the ∼5,600 genes experiencing AS at that stage (Supplemental Tables S1 and S2). ES and IR were the two major types of AS (Supplemental Tables S2 and S3). Together they contributed to 54%–61% of the total differential alternative splicing (DAS) events in maize leaves from plants exposed to higher elevated MDT compared to those at normal temperature (31°C MDT). The number of DAS events rose when plants were exposed to elevated MDT. Based on the comparison to plants at 31°C MDT, 544 and 691 DAS events were identified at developmental stage V4 (20 DAG) and V5 (27 DAG), respectively (Supplemental Table S3). At developmental stage V4 (20 DAG), increasing numbers of DASs were identified at 33°C, 35°C, and 37°C when compared to the number of ASs at 31°C MDT (Figure 1, A and B, Supplemental Tables S3 and S4). The greatest overlap of genes subjected to DAS occurred in comparing 35°C/31°C to 37°C/31°C MDT. (Note that the frequency of splicing events will be expressed throughout as the difference between the higher MDT and 31°C MDT and designated as 35°C/31°C MDT, for example). Increases were observed in nearly every type of AS event, not just on a single type of event. In comparing 37°C/31°C MDT at developmental stage V4, IRs were most abundant followed by ESs (Figure 1B, Supplemental Tables S3 and S5). At a later developmental stage (V5), fewer DASs were identified in comparing 37°C/31°C than in comparing 35°C/31°C MDT (Figure 1, C and D). It is possible that chronic exposure to the higher temperature (37°C MDT) impacts the frequency of AS. ESs were the most abundant at stage V5, followed closely by IRs (Figure 1D). Thus, increases in MDT were not selective with respect to the type of DAS, but promoted all basic types of ASs. Interestingly, few of the DAS genes were differentially expressed by elevated MDTs (Supplemental Tables S3–S6), indicating largely that transcriptional regulation and AS are independently involved in heat stress response.

Most genes had a single type of AS; however, 89 out of 679 DAS genes experienced multiple types of ASs (Supplemental Figure S2). For example, a homolog of Arabidopsis *CALCIUM UNDERACCUMULATION 1* (Zm00001d004109, CAU1), required for epigenetic silencing of FLC (Flowering Locus C), produced RNA isoforms derived from A5SS, MXE and IR. Transcripts from a SR protein gene Zm00001d040672 (SRP34) were subject to A5SSs, MXEs, IRs, and ESs. Another SR protein gene (Zm00001d047847, SR45a) produced transcripts that underwent A5SS, MXE, and ES ASs. As pointed out above, ESs and IRs were more frequent than the other three types of DAS in response to increased MDTs.

### Enrichment analysis of alternatively spliced genes in response to high MDT

Using gene ontology (GO) analysis, we found that the ∼900 events due to increasing DAS at higher MDT were enriched for three major biological processes, RNA processing, plant development and protein modification/chromosome segregation (Supplemental Figure S3 and Supplemental Table S7). Some examples of genes encoding protein kinases that were differentially spliced with increasing MDT included *MITOGEN-ACTIVATED PROTEIN KINASE KINASE KINASE 3* (*MAPKKK3*) and a *RECEPTOR-LIKE SERINE/THREONINE PROTEIN KINASE* (*NCRK*; Supplemental Figure S4A and Supplemental Table S8). Some regulatory genes involved in plant development with increased AS at elevated MDT include NF-YB1 and CONSTANS-LIKE9 protein genes. Maize plants subject to 35°C or 37°C MDT displayed clear signs of a heat shock response (HSR; Li et al., 2020). However, the only HSF that was differentially spliced in response to increased MDTs at the V4 stage was HSFTF9 (Zm00001d048041). The AS pattern for HSFTF9 in maize differed from the heat-shock induced ESs in HSF2 of rice and Arabidopsis (Sugio et al., 2009). In maize, heat-induced increases in IRs led to a greater abundance of HSFTF9 with longer 3′-UTRs. Few of the genes described above were upregulated in their expression by elevated MDTs, indicating that the response to heat for most of these genes involved qualitative changes in the pattern of RNA isoforms produced rather than quantitative changes in their overall abundance (Supplemental Figure S4B).

The trend was in the opposite direction for some other genes, in which their AS frequencies were reduced with increasing MDT. In one group of genes that encode phosphatidylethanolamine binding (PE-binding) proteins, AS frequencies declined with increasing MDT (Supplemental Figure 5A and Supplemental Table S8). The three PE-binding protein genes are homologs of flowering locus T (FT), however, they have not been shown to be orthologs, i.e. genes that support photoperiod control of flowering (Yoo et al., 2005). The three PE-binding protein genes were subject to DAS, and two had reduced DAS at higher MDT at both development stages. For these genes, the decline in AS with increasing MDT could not be entirely attributed to down regulation in expression since the genes were subject to a lesser decline in gene expression than their decline in DAS (Supplemental Figure S5B).

### AS of splicing factor and splicing regulator genes in response to increased MDTs

**Figure 2:**
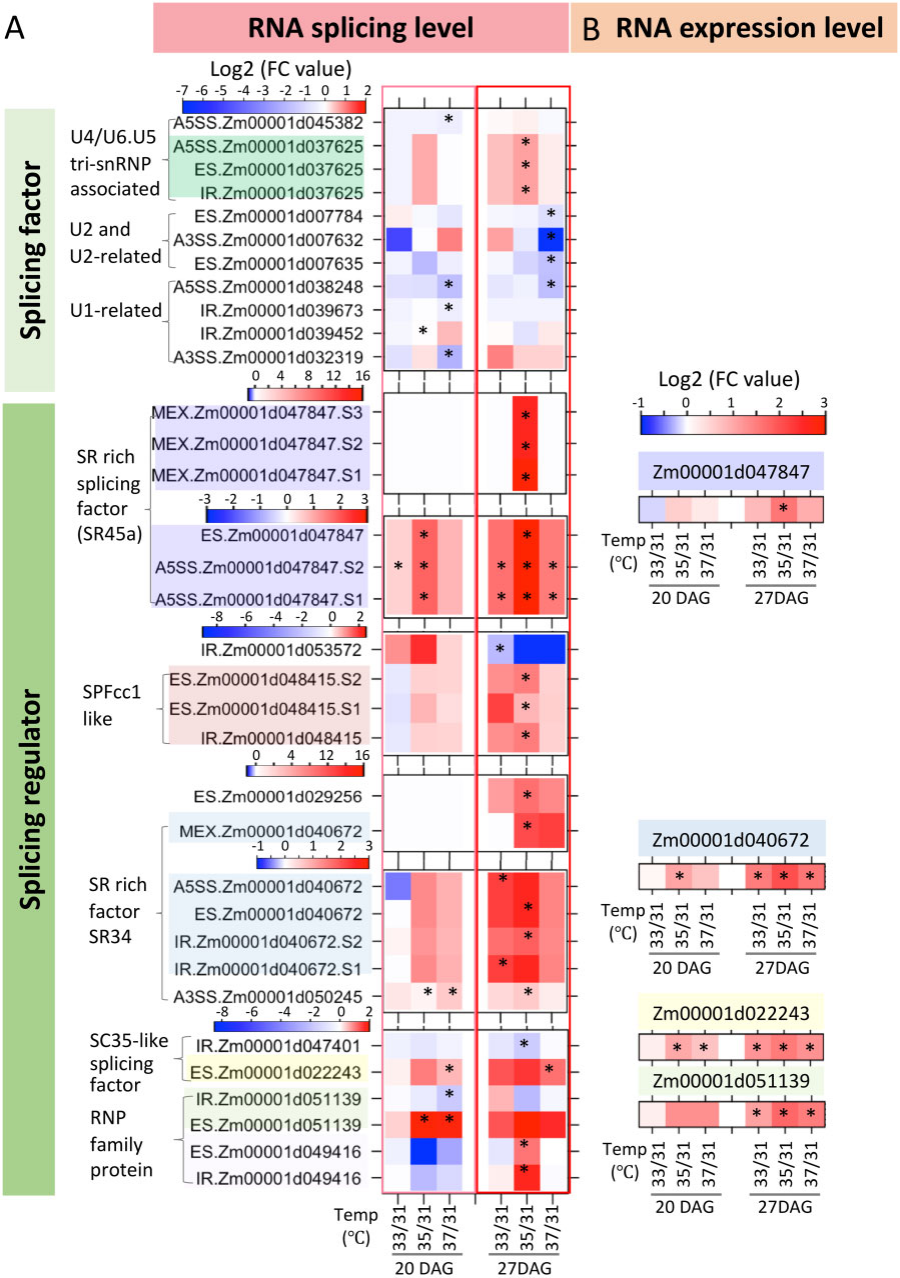
AS changes in genes involved in RNA processing. A, The heat map shows changes in AS in response to increased MDTs compared to 31°C MDT. Each column in the heat map represents changes in abundance of a particular type of AS. Heat map has been divided into two parts to separate the genes encoding splicing factors from those encoding splicing regulators. Note that certain genes have several different types of ASs (S1 means site 1, S2 site 2 and so forth). The color background in the gene IDs is used to show that the group of ASs belongs to a specific gene. * indicates significant differential splicing when compared to the splicing at 31°C MDT. The log2 fold change (FC value) is the ratio of DAS events when comparing the number of events at the higher MDTs to that at 31°C MDT. DAS events refer to ES/MXE/intron retained/alternated 5/3′ splice site when AS in that region is ES/MXE/IR/A5SS/A3SS, respectively. Thresholds of |Δψ| > 5% and FDR ≤1% were used in assessing differential splicing. B, Heat map for gene expression changes. Shown are four genes from A that have significant gene expression changes. Differentially expressed genes were identified with Q value ≤ 0.05 and absolute log_2_ FC (high MDT compared to 31°C MDT) more than 1. * indicates significant differential expression when compared to the expression at 31°C MDT.

Some of the genes subject to DAS with increasing MDT encode splicing regulators themselves (Figure 2A and Supplemental Table S8). The factors can be classified as those that encode core spliceosome components (hereafter, splicing factors) and those that encode splicing regulators (Ru et al., 2008). The AS of genes that encode splicing regulators were the most highly upregulated with increasing MDTs. The greatest increase in AS with increasing MDT occurred in comparing 35°C/31°C MDT at vegetative stage V5 (27 DAG), rather than comparing 37°C/31°C MDT. As before, this may be due to the fact that plants subjected to 37°C MDT were weaker because of longer exposure to the more stressful conditions.

To determine if increasing DAS at higher MDTs is simply due to greater abundance of pre-mRNAs, we compared the expression levels of the splicing factor/splicing regulators genes at the different MDTs. Only four genes encoding splicing regulators were moderately upregulated with increased MDT (Figure 2B). The expression of genes encoding a SR45 family member, SR45a (Zm00001d047847), a mRNP protein (Zm00001d051139), a SCL-subfamily SR protein SCL33 (Zm00001d022243), and a SR subfamily protein SR34 (Zm00001d040672) were all upregulated by elevated MDT at 20 DAG and 27 DAG less than eight-fold (log2FC < 3). However, the fold change in AS for these genes was much greater than heat-induced expression. For example, the heat-induced expression of SR45a (Zm00001d047847) and SR34 (Zm00001d040672) was about seven-fold, however, the heat induced AS at certain sites for these genes was more than 1,000-fold (log2FC > 10). For other splicing regulator genes, the increases in AS at higher MDTs were not accompanied by increases in their expression levels.

**Figure 3:**
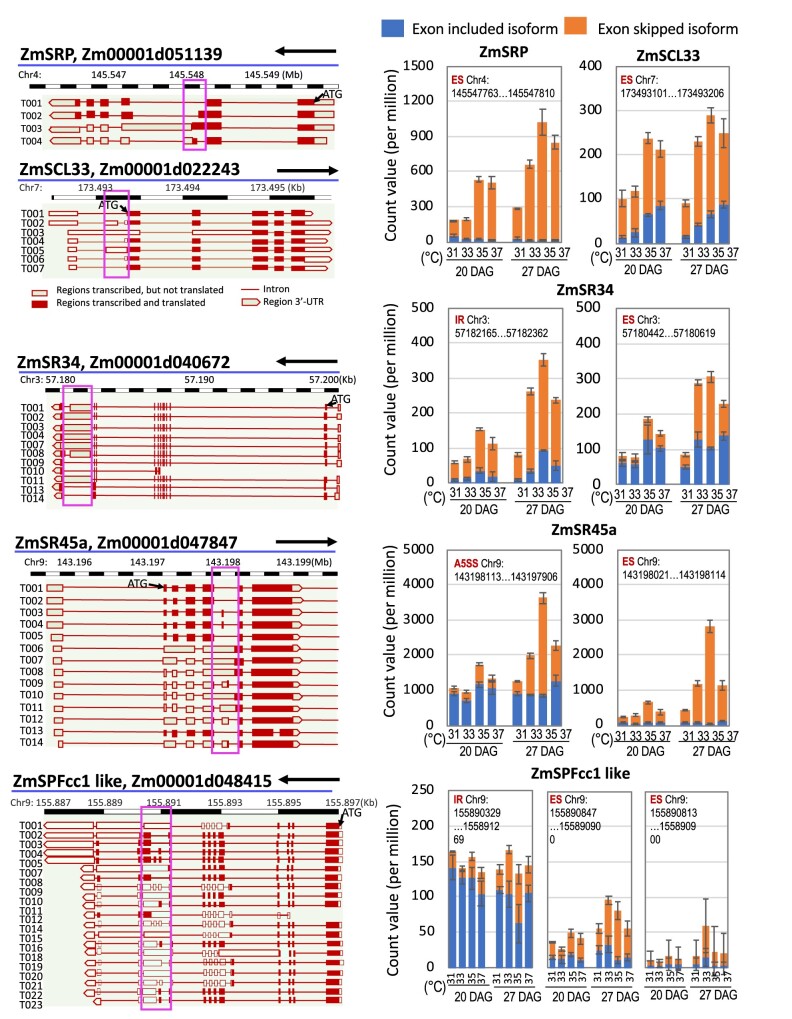
AS changes in genes involved in RNA processing. Gene models (left) and stacked bar graphs (right) show AS changes in splicing regulator genes in response to increased MDTs. Each graph shows a different type of AS and the numbers represent the chromosomal positions (in the boxes in the gene structures on the left) in which read counts were tallied for RNA isoforms with exons included (within the red box) versus RNA isoforms with exons skipped. Blue bar indicates exon included/intron removed/normal 5/3′ splice site isoform when AS in that region is SE/IR/A5SS/A3SS. Orange bar indicates exon skipped/intron retained/alternated 5/3′ splice site isoform when AS in that region is SE/IR/A5SS/A3SS, respectively. Bars represent the mean from the RNAseq data of three biological replicates. Errors = sd.

Splicing regulator proteins, such as the SR proteins, participate in the initial steps of AS and interact with U1, U2 complexes and U4/U6.U5 tri-snRNP associated complexes (Tanabe et al., 2009). Splicing regulators contribute to AS, because splice-site selection is determined not only by splicing factors, i.e. core spliceosomal components, but also to a large extent by other RNA-binding proteins, such as the splicing regulators, which bind to *cis*-regulatory elements located in either introns or exons, thereby activating or repressing splicing (Laloum et al., 2018). Five splicing regulatory genes that showed differential patterns of AS with increasing MDT were selected for further analysis (Figure 3, Supplemental Figures S6 and S7). They include SRP (Zm00001d051139), SCL33 (Zm00001d022243), SR34 (Zm00001d040672), SR45a (Zm00001d047847), and SPFcc1-like (Zm00001d048415). We analyzed the splicing pattern for these genes at different MDTs in leaf samples at 11:30 ZT when temperatures reached their highest level for the day (Figure 3, Supplemental Figures S6 and S7). For SR34 (Zm00001d040672), the frequency of IRs increased with higher MDTs resulting in transcripts predicted to have longer 3′-UTRs. The RNA isoforms produced at higher MDT for SRP and SPFcc1-like are predicted to encode longer proteins. At lower MDT, the major RNA isoforms for these two genes have a long exon with in-frame PTCs. In the case of SR45a, higher MDTs led to the production of RNA isoforms capable of encoding full-length (FL) proteins starting from an early translation start site (TSS). In the SR45a RNA isoforms produced under lower MDT conditions, the ORF beginning from the same TSS is blocked by several PTCs (Supplemental Figures S8 and S9). However, a sizable ORF is open from a downstream ATG. Cognizant of the concerns raised by Brown et al. (2015), we cannot say that because an upstream ORF is blocked, that a downstream ORF will be used. Nonetheless, if the transcripts produced at lower MDT are translated to produce SR45a proteins, then the downstream ORF would have to be used. In the case of SCL33, the ASs produced at higher MDT led to changes in the 5′-UTR. All of the other genes in this group except for SCL33 have enhanced production at elevated MDTs of RNA isoforms capable of producing longer splicing regulators. (Note that the reads are tallied for ASs in a region of a gene where the indicated ASs have occurred. The numbers on each graph represent chromosomal positions in which reads were tallied as a splice leading to a predicted longer protein or an AS. As a result, the number of reads for FL splicing forms for a particular gene, such as SR34, will differ because reads are assessed in different regions of the gene.)

### Longer SR45a isoforms increased in response to increased MDTs

**Figure 4:**
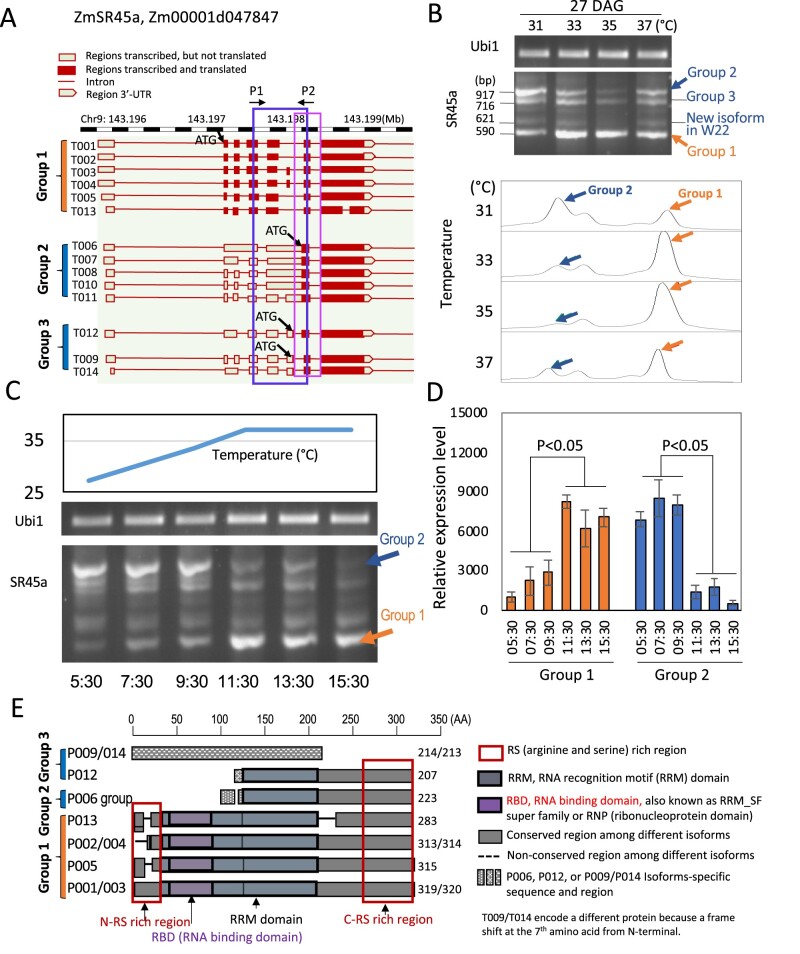
AS changes in SR45a in response to increased MDTs. A, SR45a RNA isoforms predicted from the sequence of maize B73 (V4 version). Box indicates the region in which amplicons were generated and in which heat-induced ASs occur as ascertained from the RNAseq data. B, Amplicon patterns in upper panel show changes in SR45a RNA isoforms in response to increased MDTs. V4 and V5 plants from chambers with different MDTs were sampled for RNA analysis at ZT 11:30. Orange arrows indicate amplicons representing Group 1 isoforms that increase with increasing MDT, while blue arrows indicate Group 2 isoforms that decline under these conditions. Ubiquitin 1 represents a loading control. Lower panel shows scans of the gel in the upper panel. C, Time course of daily changes in SR45a RNA isoforms in response to different MDTs. Plants were sampled every 2 h for analysis. All three replicates showed the same pattern and one replicate are displayed here. D, For the bar graph, the relative expression levels of certain bands were quantified with Image J (https://imagej.nih.gov/ij/). Values are the means of three replicates ± sd, *P* < 0.05 indicate significant differences between comparisons in each group isoform (*P* < 0.05, one-way ANOVA). E, Maps of the proteins predicted from different SR45a isoforms. The predicted protein isoforms highlighted in orange increase in response to increased MDTs and the ones highlighted in blue decrease. Note the presence or absence of the N-terminal RS and RBD domains in the various isoforms.

The SR protein gene ZmSR45a (Zm00001d047847) was selected for further study because this gene had the highest frequency of AS among the splicing regulator genes. This gene is one of six genes annotated as SR45a in maize (plants.ensembl.org/Zea_mays/Info/Index). The gene stands alone among its SR45a cohorts in maize and has no homologs in orthologous regions of the Arabidopsis or rice genomes (Supplemental Figure S10). Unlike Arabidopsis SR45a, which is not closely related in sequence of maize SR45a and is primarily expressed in carpels and sepals, ZmSR45a (Zm00001d047847) is highly expressed in leaves (Supplemental Figure S11; plants.ensembl.org/Zea_mays/Info/Index). The maize gene is composed of seven exons, and 14 RNA isoforms have been described for this gene in inbred line B73 (Figure 4A; https://plants.ensembl.org/Zea_mays/Info/Index). The gene has an untranslated first exon followed by a large intron. Most of the AS RNA forms are generated by splicing variations in the group of smaller exons and introns that follow the large intron. The splicing patterns for SR45a transcripts were monitored by semiquantitative RT-PCR because amplicons of the different forms are of different sizes that can be separated on gels. At developmental stage V5 (27 DAG), 4-5 different bands could be easily discerned on gels (Figure 4B, upper panel). The amplicons were divided into three groups based on the structure of the RNA isoforms they represent (Figure 4A). Amplicons of about 917 bp were found by sequencing analysis to represent group 2 isoforms (T006, T007, T008, T010, and T011). The abundance of these amplicons decline at higher MDT, 35°C and 37°C (Figure 4B, lower panel). The isoforms that these amplicons represent have A3SS and ES2-5 ASs. Another group of amplicons of about 590 bp represent group 1 RNA isoforms (T001-T005 and T013), which have the longest predicted ORF (although T013 has an extra intron in the terminal exon). Amplicons representing group 1 transcripts increase in abundance with temperature, particularly at 33°C and 35°C MDT (Figure 4B, lower panel). The increase in group 1 SR45a transcripts and the decline in group 2 SR45a transcripts were validated by reverse transcription quantitative PCR (RT-qPCR) using isoform specific primers (Supplemental Figure S7A). Another group of isoforms, group 3, is composed of amplicons representing T009/T014 and T012. The difference between T009/T014 and T012 is only a single base deletion due to an A5SS.

Changes in SR45a mRNA isoforms were tracked during the simulated day. Samples from the first fully expanded leaves of V4 plants grown at the 37°C MDT were collected every 2 h. RNA was extracted and RNA patterns were analyzed as before. During the virtual morning, the amplicons representing group 2 RNA isoforms declined, particularly around 11:30 ZT when the temperature reached its peak at 37°C MDT (Figure 4, C and D and Supplemental Figure S7C). On the other hand, the amplicons representing group 1 isoforms increased during that same period. Group 2 SR45a transcripts that decline in the virtual morning, if translated would be predicted to encode shorter proteins missing the N-terminal 117 amino acids including the N-terminal RS domain and RNA binding domain (RBD; Figure 4E;Supplemental Figure S12). The Group 2 RNA isoforms such as T006 are nonproductive having long exon sequences containing multiple short upstream ORFs (Supplemental Figures S8 and S9) making it uncertain whether the major downstream ORF of T006 is translated and difficult to understand how the transcript with all of its PTCs avoids NMD.

The AS pattern for maize SR45a differs from that of Arabidopsis (Supplemental Figure S13) in which the N-terminal 30 amino acids and the C-terminal RS domain are spliced out (Tanabe et al., 2009). The loss of the N-terminal 30 amino acids in AtSR45a appears to be of little consequence to the predicted protein. However, in maize, the loss of 117 amino acids from the N-terminus by AS is predicted to have a greater impact because the loss eliminates certain splicing factor functionalities (Figure 4E). Group 1 amplicons, which represent transcripts with increased abundance in the afternoon, could encode larger proteins that retain both RS domains and the RBD. However, the N-terminal RS domain was slightly different in some of these Group 1 isoforms because of a A3SS AS in exon 4, which results in four to five amino acid changes (Figure 4, A and E; Supplemental Figure S12).

### Performance of SR45a RNA isoforms in RNA splicing assays

**Figure 5:**
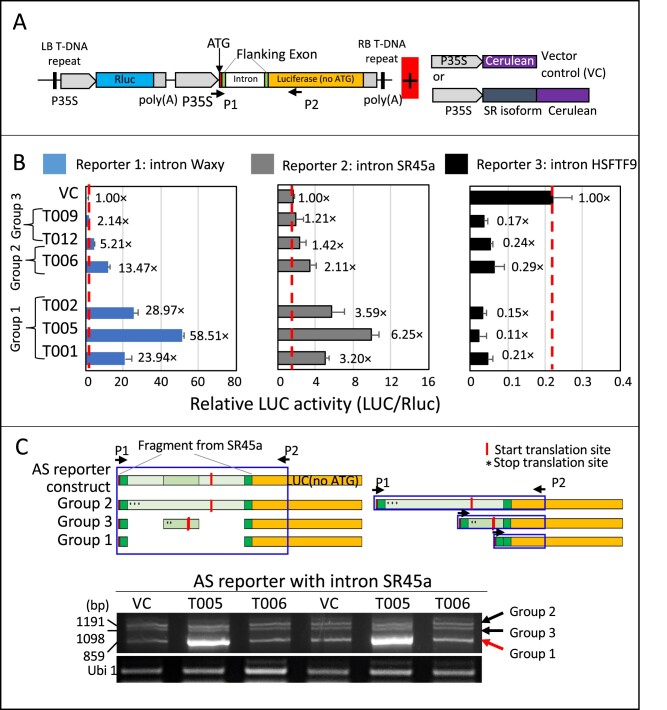
Splicing efficiency of the different SR45a protein isoforms. A, Diagram of the dual-luciferase reporter used to assess RNA splicing efficiency and the experimental design of the splicing efficiency assay using the maize mesophyll protoplast system. The reporter consists of an intron, linked to the firefly luciferase gene. Splicing out of the intron permits expression of the firefly luciferase reporter. To evaluate RNA splicing efficiency of different SR45a protein isoforms, the activity of the firefly luciferase reporter is assessed in protoplasts co-transfected with the luciferase reporter and recombinant forms of SR45a. B, Splicing efficiency of different SR45a protein isoforms using the AS reporter with introns from the Waxy gene, SR45a, and HSFTF9 (heat stress factor) gene. Splicing efficiency activity is expressed as the relative activity of firefly luciferase versus Renilla luciferase. Values are the means of the four replicates ± sd. Values indicate the fold increase in firefly luciferase activity resulting from the expression of the SR45a isoforms. Empty pAN578 (the backbone of SR gene overexpression constructs) was used as a control (VC, one-fold). C, Splicing activity of the different SR45a isoforms assessed by RT-PCR using the intron from the SR45a gene itself in the context of the luciferase AS reporter described above. Amplicons from the products of splicing correspond to Group 1, 2, and 3 SR45a transcript isoforms. The splicing patterns were generated in protoplasts co-transfected with the AS luciferase reporter and different SR45a isoforms (T005, T006, and VC, the empty pAN587 control). Box indicates the region in which amplicons were generated and arrows indicate the positions of the primers used. Co-transfection with SR45a predicted to be derived for RNA isoform T005 (a Group1 SR45a isoform) increased RNA splicing efficiency.

If the longer ORFs in the identified RNA isoforms were translated, we wanted to know whether the proteins encoded by the different RNA isoforms would be effective in their support of RNA splicing. To address that question, we set up a RNA splicing assay system in maize mesophyll protoplasts (Figure 5A). The assay assesses the ability of the different protein isoforms to splice out an intron from a model RNA substrate in order to activate the expression of a reporter gene, firefly luciferase (Supplemental Figure S14). Therefore, recombinant forms representing the protein coding regions of Group 1, 2, and 3 isoforms were co-transfected with the luciferase constructs in maize protoplasts, respectively. Splicing ability of the recombinant forms was tested using three model substrates with introns from the waxy gene, the SR45a gene itself and from HSFTF9. The latter two were identified as showing greater changes in RNA isoform patterns at elevated MDTs. We found that the proteins produced from Group 1 SR45a RNA isoforms enhanced the splicing of the waxy and the SR45a introns, and that the proteins produced from the T005 RNA isoform outperformed them all in splicing efficiency (Figure 5, B and C). To test the effect of the upstream regions on the translation of the SR45a, especially the small uORFs in the upstream of group 2 SR45a transcripts, two different group 2 SR45a full transcripts and one group 1 SR45a full transcripts with their upstream region were amplified (Supplemental Figure S9). We found that the FL group 2 transcripts with the small uORFs fared no better in encoding proteins for splicing the waxy and the SR45a introns than the transcripts encoding the coding region alone (Supplemental Figure S15). Thus, the SR45a RNA isoforms, which were upregulated in the afternoon, are capable of encoding proteins that are more efficient in splicing the two model constructs. On the other hand, expression of the proteins encoded by Group 1 RNA isoforms appeared to repress the endogenous level of AS for HSFTF9. Repression of AS was the case even for the proteins encoded by Group 2 and 3 isoforms that fail to enhance the splicing of introns from the waxy and SR45a substrate. Thus, SR45a appears to be an important factor either in the enhancement or in the repression of stress-induced AS.

### Role of conserved SR45a domains in RNA splicing

**Figure 6:**
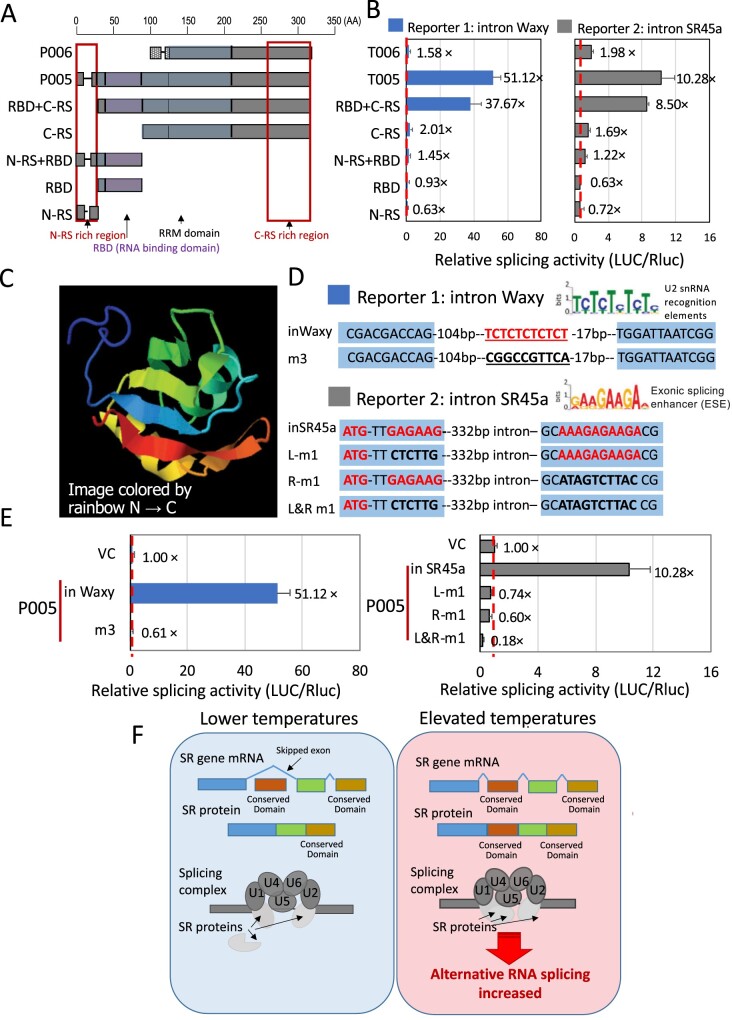
Splicing efficiency of SR45a domain and working model of heat stress-induced SR genes AS. A, Maps of the proteins predicted to be translated from different SR45a RNA constructs in pAN578. B, Splicing efficiencies of different SR45a truncated forms were assessed using the AS reporter with introns from the Waxy gene and SR45a gene. Splicing efficiency of the constructs is expressed as the relative activity of firefly luciferase versus Renilla luciferase and is shown in comparison to the splicing efficiencies contributed by the proteins produced from Group 1 P006 and Group 2 P005 RNA isoforms. Values are the means of the four replicates ± sd. Values indicate the fold change (VC) in luciferase activity by coexpressing different SR45a forms. Empty pAN578 (the backbone of SR gene constructs) was used as an empty vector control (VC, one-fold). C, The N-terminal RBD of P005 group proteins, but lacking in the P006 group. The domain is ∼90 amino acids in length and consists of four beta-sheet strands packed against two alpha-helices. 3D structure of the P005 RBD was predicted using Phyre2 (http://www.sbg.bio.ic.ac.uk/∼phyre2/html/page.cgi?id=index). D, Candidate motifs for the binding by the RBD to the RNAs of the model substrates. Pyrimidine-rich intron binding motif and a M3 mutant are shown for the waxy substrate and a purine-rich exon binding motif and two M1 mutations are shown for the SR45a substrate. E, Splicing efficiency of the nonmutant substrates and the mutants is expressed as the relative activity of firefly luciferase versus Renilla luciferase. Values are the means of the four replicates ± sd. Values indicate the fold increase contributed by expression of SR45a isoforms. Empty pAN578 (the backbone of SR gene overexpression constructs) was used as a control (VC, one-fold). F, Working model for heat induced AS. Global AS is induced by increasing MDT. The SR genes, which are splicing regulators, are major targets of differential AS. With a higher afternoon temperature, AS produces RNA isoforms capable of encoding functionally efficient splicing regulators with the potential to enhance AS for other genes.

The differences between proteins that are capable of being produced by the two groups of SR45a isoforms lie in the N-terminal RS domain that promotes its interaction with other splicing factors and in the RBD which stabilizes the binding of the factor to ssRNA (Figure 4E; Supplemental Figure S12). To identify which domain contributes most to the different splicing efficiency of SR45a, five DNA constructs that lacked the different domains were co-transfected with the AS reporter (Figure 6A). (Note: these constructs do not represent natural RNA isoforms; however, they were produced in order to further dissect SR45a to determine which of the N-terminal domains are required for splicing.)

The deletion of the N-terminal RS domain and the RBD, similar to what could be produced from the T006 isoform, abolished RNA splicing (Figures 4E and 6B) indicating that one or both of the N-terminal RS domain and the RBD were essential for splicing the model substrates. However, when only the N-terminal RS was deleted, splicing activity was only slightly reduced. Therefore, the elimination of the coding capacity for the RBD in Group 2 isoforms, and not the N-terminal RS domain, is largely responsible for their diminished splicing activity. The RBD in SR45a is ∼90 amino acids in length and is predicted to be a four-stranded beta-sheet packed against two alpha helices (Figure 6C; Supplemental Figure S16).

We also attempted to determine what characteristics of the target RNA substrate are required for splicing in the protoplast system. As a control, we altered the polypyrimidine tract in the waxy intron that likely serves as the recognition site for branch point factors involved in lariat formation (Ruskin et al., 1984; Frendewey and Keller, 1985; Reed and Maniatis, 1985; Garcia-Blanco et al., 1989; Reed, 1989; Figure 6D). Substitution of the polypyrimidine-rich TCTCTCTCTCT sequence with CGGCCGTTCA effectively eliminated the splicing of the waxy intron by SR45a P005 (Figure 6E). This showed that the splicing assay truly assesses RNA splicing because it depends on the integrity of the branch point and that some other uncharacterized activity in the protoplast system is not responsible for the expression of the luciferase reporter. Xing et al. (2015) identified RNA motifs as binding sites for AtSR45 (a different SR protein with calculated molecular mass of approximately 45 kDa, not to be confused with SR45a) based on RIP-seq experiments (RNA-protein immunoprecipitation sequencing experiments). The binding sites were G/A rich motifs with features typical of exonic splicing enhancers (ESE). The motifs were positioned within exons at the intron boundaries (Figure 6D). To test if maize SR45a uses these motifs for the RNA splicing, reporters with or without different mutations in these ESE-like RNA motifs were co-transfected with SR45a T005 in the maize protoplast system. Mutations in these motifs severely reduced the splicing efficiency of SR45a P005, especially the mutation in both the left and right ESE-like RNA motifs in the SR45a reporter (Figure 6E). This indicates that SR45a recognizes and binds conserved RNA motifs to help catalyze RNA splicing. Thus, both *cis*-acting ESE-like RNA motifs and *trans*-acting RBDs in SR proteins are essential for AS by the spliceosome.

## Discussion

In this study, we found that elevated MDTs had global effects in promoting AS. The frequent targets of AS were genes encoding RNA splicing regulators, especially SR proteins. We focused on one of the plant-specific SR protein genes in maize, SR45a, the splicing of which was enhanced at elevated temperature in the context of diurnal changes in daily temperatures. At lower daily temperatures in the virtual morning of a hot summer day, SR45a RNA isoforms were produced that were replete with short upstream ORFs, but if the longer downstream ORFs were translated, they would lack predicted N-terminal RS and RBDs. Later in the day, other RNA isoforms are produced which would be predicted to encode fully functional SR45a forms with both RS domains and the RNA binding and RRM domains. The afternoon RNA isoforms would encode proteins that are efficient in splicing model substrates, such as an intron from the waxy gene and another from SR45a itself. If translated, the morning RNA isoforms would encode proteins that are less efficient, not because of the loss of the N-terminal RS domain, but because of the loss of the RBD. Hence, in maize, progress through the day leads to shifts in the production of alternatively spliced SR45a isoforms that are capable of producing splicing regulators more efficient in splicing (Figure 6F).

In the model substrates used to test the splicing efficiency of the SR45a isoforms in the maize protoplast system, two RNA motifs were identified as binding sites for AS factors. The sites were the two G/A rich ESE-like motifs in the exons at the boundaries of the intron in the SR45a substrate. Mutations in these RNA motifs abolished the splicing efficiency of SR45a. This indicated that SR45a recognizes and binds to conserved RNA motifs that enhance spliceosome catalyzed AS.

It is difficult to understand why plants make the RNA isoforms at lower MDTs only capable of encoding inefficient splicing regulators or blocked in making any splicing regulators by small upstream ORFs. It is important to point out that we have taken into account the issues raised by Brown et al. (2015) in that we do not assume that a downstream ORF is translated in an alternatively spliced form simply because upstream ORFs have been blocked by PTCs. Several of the RNA isoforms, such as SR45a T006, have retained introns with PTCs situated >50 bp upstream from the 3′ splice site and should be candidates for NMD (Supplemental Figures S8 and S9). Yet they seem to accumulate during the virtual morning/lower MDTs. It is of interest that Kalyna et al. (2012), who compared the abundance of transcripts in NMD defective mutants compared to wild type, found that not all transcripts with IRs containing PTCs were targets for NMD in Arabidopsis. Such transcripts may be sequestered in the nucleus and may not make it out to the translation machinery where they might subject to NMD. Perhaps the fact that the RNA isoforms capable of encoding shorter proteins decline after virtual midmorning may be an indicator that NMD activity commences at this time. It is possible that the downstream ORF encoding the inefficient splicing regulator is not translated at all, in which case the consequences of AS would be about the same whether the RNA isoform was translated or not.

The splicing of other SR45 genes has been described in Arabidopsis, however, these genes are not closely related to the maize gene in this study (SR45 named because of the calculated molecular mass for these proteins is ∼45 kDa), and the patterns of splicing are also different. In Arabidopsis, Gulledge et al. (2012) found that an exon-skipped RNA variant of SR45a (At1g07350.1) encoded a FL protein while an unskipped form lacked a C-terminal RS region (Supplemental Figure S13). Heat treatment in the Gulledge study and high light in another study by Tanabe et al. (2009) tended to favor the accumulation of the exon skipped form. Zhang and Mount (2009) analyzed the effects of splicing of another SR45 gene in Arabidopsis (At1g16610) which created an isoform in which a single amino acid in exon 7 of SR45.1 was replaced by eight amino acids. They found through complementation analysis that the isoforms appeared to have different functions in that SR45.1 affected petal phenotypes while SR45.2 complemented root growth phenotypes.

AS has been reported in other plants under stress conditions. Matsukura et al (2010) described the stress-inducible splicing of OsDREB2B in rice. Under non-stress conditions, a shorter non-functional form is produced, and in response to stress conditions a longer form is made. The non-functional form is not a precursor of the functional form, rather the functional form is produced by AS. The OsDREB2B gene is composed of three exons and under normal conditions, splicing includes all three exons, but encodes a shorter protein. Under stress conditions, ES eliminates exon 2 from OsDREB2B, which connects exon 1 in frame with a long open frame in exon 3. Thus, by the elimination of exon 2, the OsDREB2B gene transcript encodes a longer protein. Matsukura et al. (2010) went on to show that the longer protein was more active as a transcription factor in an Arabidopsis transient expression system. Thus, in response to heat, drought and high salinity conditions, OsDREB2B transcripts are alternatively spliced giving rise to a functional form of this stress transcription factor.

Since AS increases with MDT in maize, it is of interest to know whether AS is involved in the HSR, which is a major contributor to heat stress tolerance in plants (Vierling, 1991). Zhang et al. (2020) observed slight increases in the alternatively spliced forms of ZmHsf04 and ZmHsf17 following heat shock (42°C) in maize. In Arabidopsis, AS is responsible for the induction of at least one of the heat shock factors, *HsfA2*. *HsfA2* contains a single 324 nt intron that is spliced out at 22°C leading to the production of a FL *HsfA2* transcript (Sugio et al., 2009). Elevated temperature conditions (37°C) produce a RNA isoform, *HsfA2-II*, that includes within the RNA transcript a 31 nt cryptic miniexon that lies within the *HsfA2* intron. The minexon has a PTC, which results in the degradation of the RNA by NMD. An additional splice variant *HsfA2-III* is produced because of a cryptic 5′ splice site in the intron. This splice site is used at higher temperature (42°C–45°C), reducing the production the *HsfA2-II* variant (Liu et al., 2013). *HsfA2-III* encodes a truncated protein, S-HsfA2, which can bind to the *HsfA2* promoter, upregulating HsfA2 expression, and constituting a positive autoregulatory loop.

Another gene that is subject to temperature dependent AS is *FLOWERING LOCUS M* (*FLM*), a component of the thermosensory flowering pathway in Arabidopsis (Capovilla et al., 2017). FLM acts as a “thermometer,” measuring moderate changes in ambient temperature. FLM encodes two RNA isoforms, *FLM-β* and *FLM-δ*, which can be translated into two protein isoforms, differing by the inclusion of either the second or third exon, (Jiao and Meyerowitz, 2010). The abundance of *FLM-β* relative to *FLM-δ* changes in response to temperature (Balasubramanian et al., 2006). *FLM-β* is down-regulated with increases in temperature, signifying that *FLM-β* and *FLM-δ* function differently in the control of flowering by ambient temperature (Balasubramanian and Weigel, 2006).

Of recent interest is whether or not RNA splicing in plants is cotranscriptional. The work of Li et al. (2020) and Zhu et al (2020) demonstrate that cotranscriptional splicing is widespread in Arabidopsis despite the fact that plant genes are shorter than their mammalian counterparts, which might lead one to think that the incidence of cotranscriptional splicing would be much lower in plants. Nonetheless, the considerable extent of cotranscriptional splicing in plants is relevant to AS because the environment in which splicing occurs can be influenced by chromatin structure and histone modifications. Perhaps, this is the key as to why some plant genes are more subject to AS while others are not.

## Experimental procedures

### Plant material

Maize (*Zea mays*) inbred line W22 was used in this study. This inbred line was developed at the Wisconsin Agricultural Experiment Station and served as one of the platforms for maize genetics (Brink et al., 1968). In this study, the middle part of the newest, fully expanded leaf from plants at the V4 (20 DAG) and V5 (27 DAG) stages were collected for RNAseq analysis and RT-PCR analysis. The leaf from 10 DAG seedlings was used for maize protoplast isolation and transient expression.

### Plant growth conditions

Plant growth conditions were the same as described in Li et al. (2020). Briefly, seeds of maize W22 were geminated in soil in small pots with 13 h light/11 h dark at 26°C. For experiments in the Enviratron, seven DAG plants were transplanted in nine-inch pots and randomly placed into 12 set positions within each chamber. The eight growth chambers in the Enviratron were used to simulate four different environmental conditions, i.e. with duplicate chambers for each condition. The daytime temperatures were ramped up over 6 h to a MDT of 31°C, 33°C, 35°C, or 37°C (Supplemental Figure S1). Nightime temperatures were ramped down over 8 h to 10°C below the MDT. Light intensities in a 16 h/8 h photoperiod cycle were also ramped up to 1200 µmol m^−2^ s^−1^ (measured 60 cm from the light source) or down over 2 h periods to simulate dawn and dusk. Soil water potential was maintained at 50% VWC (volumetric water content) and relative humidity was held at 60%. Leaf samples from plants under different growth conditions (different MDTs) were collected for transcriptome analysis at the V4 (20 DAG) and V5 (27 DAG) developmental stages.

For experiments utilizing maize protoplasts, seeds of maize W22 were geminated in soil in small pots with 13 h light/11 h dark at 26°C until 10 DAG, then leaves from plants were collected for protoplast isolation.

### Transcriptome, alternative spicing, quantitative RT-PCR and semiquantitative RT-PCR analyses

RNA was extracted from small samples (0.1 g) of leaf lamina (avoiding the midrib) obtained from the middle of the first fully expanded leaves. RNA was isolated using a Plant RNeasy Mini Kit and treated with DNase (Qiagen, www.qiagen.com/us/) according to the manufacturer’s instructions. RNA quality and quantity were determined by the UV absorbance spectra, gel electrophoresis, and Agilent 2000 BioAnalyzer (http://www.agilent.com).

For quantitative RT-PCR and semiquantitative RT-PCR, 500 ng total RNA were used for the cDNA synthesis (iScript cDNA Synthesis kit (Bio-rad, www.bio-rad.com)), which in turn was utilized as template for quantitative RT-PCR and semiquantitative RT-PCR analyses. Quantitative RT-PCR (RT-qPCR) was performed with StepOnePlus™ (Applied Biosystems, https://www.biosciences.ie/applied-biosystems) using a PowerTrack™ SYBR Green Master Mix (Thermo Fisher Scientific, A46012) according to the manufacturer’s instructions. Relative gene expression levels were calculated using the 2^−ΔΔCt^ method (Livak and Schmittgen, 2001) and employing maize *ubiquitin*1 (*Ubi*1) as a standard. Primers used in this study are listed in Supplemental Table S9. Three biological replicates were used for expression analysis of different RNA transcript isoforms.

For RNAseq analysis, poly A RNA isolation, cDNA library construction, sequencing, and primary bioinformatics analysis were performed by BGI Tech Solutions Co., Ltd. (Beijing Genomic Institute (www.genomics.org.cn, BGI)) and as described in Li et al. (2020). Briefly, poly A RNA was isolated using oligo(dT)-attached magnetic beads and fragmented at elevated temperature with NEBNext RNA fragmentation buffer (New England BioLabs). First-strand cDNA was generated using random hexamer-primed reverse transcription, followed by second-strand cDNA synthesis. cDNA ends were repaired by A-Tailing followed by adding RNA Index Adapters. The cDNA fragments were amplified by PCR, and the products purified by Ampure XP Beads (Beckman Coulter). The products were validated for quality control using an Agilent Technologies 2100 bioanalyzer. The double stranded PCR products were heat denatured and circularized using splint oligos (Diegelman and Kool, 2000). The single strand circular DNAs (ssCir DNAs) were amplified with phi29 (Blanco et al., 1989) to make DNA nanoballs (DNBs; Porreca, 2010). DNBs were loaded into the patterned nanoarray and single end 50 base reads were generated on BGIseq500 platform (BGI-Shenzhen, China).

Sequencing data were filtered with SOAPnuke (v1.5.2, github.com/BGI-flexlab/SOAPnuke) by removing reads (1) containing sequencing adapters, (2) in which more than 20% of the bases have a quality score <5 or, (3) in which 5% or more of the bases are unknown (represented by N). Clean reads were stored in FASTQ format and mapped to the reference maize B73 genome using HISAT2 (v2.0.4, http://www.ccb.jhu.edu/software/hisat/index.shtml) and then used for quality control, visualization of the alignment and new transcript prediction. Bowtie2 (v2.2.5, http://bowtiebio.sourceforge.net/%20Bowtie2%20/index.shtml) was used to align the clean reads to the reference coding gene set (transcripts (cDNA), plants.ensembl.org/info/website/ftp/index.html) and then gene expression levels were calculated by RSEM (v1.2.12, github.com/deweylab/RSEM). Differential expression analysis was performed using the DESeq2 (v1.4.5, www.bioconductor.org/packages/release/bioc/html/ DESeq2.html) with Q value ≤ 0.05.

After alignment with the reference genome, rMATS (Shen et al., 2014; v3.2.5, http://rnaseq-mats.sourceforge.net) was used to detect AS in the samples. Five major types of AS were detected including ES, IR, A5SS, A3SS, and MXE. rMATS is a statistical method for robust and flexible detection of differential AS from replicate RNA-Seq data. rMATS uses a likelihood-ratio test to calculate the *P* value and false discovery rate (FDR) that the difference in the isoform ratio of a gene between two conditions exceeds a given user-defined threshold. First, the **IncLevel1** (denoted as ψ, Inclusion Level) and **IncLevel2** from two conditions is calculated as such: ψ = (I/LI)/(I/LI + S/LS) where: ψ = Inclusion Level (**IncLevel1**), I = number of reads mapped to the exon inclusion isoform (**IC_SAMPLE_1**), S = number of reads mapped to the ES isoform (**SC_SAMPLE_1**), LI = effective length of the exon inclusion isoform (**IncFormLen**), LS = effective length of the ES isoform (**SkipFormLen**). **IncLevelDifference** = **IncLevel1** - **IncLevel2.** rMATS uses a likelihood-ratio test to calculate the *P* value for the difference in the mean ψ values between two sample groups. The FDR was obtained by using the Benjamini Hochberg method, and the threshold of |Δψ| > 5% and FDR ≤1% were used to judge AS. Besides that, the different ASs (ES/MXE/intron retained/alternated 5/3′ splice site when AS in that region is ES/MXE/IR/A5SS/A3SS, respectively) in each group (high MDT/31°C) were compared to obtain the gradient comparison (33/31°C, 35/31°C and 37/31°C) of ASs, especially when FDR and |Δψ| did not meet the criterion of DAS.

### Splicing efficiency analysis by using maize protoplast transient assay

Transient expression assays were performed with maize mesophyll protoplasts as described by Sheen (2001) except W22 inbred lines were used as a source for protoplasts. For splicing efficiency analysis, two different groups of constructs were used. The first was used to overexpress different SR RNA isoforms. Different SR45a RNA isoforms or different SR45a truncated forms were amplified from cDNA of maize W22 (Supplemental Figures S17 and S18), sequenced and then inserted into the plasmid pAN578 (http://nebenfuehrlab.utk.edu/markers/vectors.htm) so that the expression of the SR45a gene isoform is driven by the 35S promoter. The second group is composed of the splicing reporters based on the modification of plasmid pGreen II 8000 (SnapGene, www.snapgene.com/resources/plasmid_files/plant_vectors/). The pGreen II 8000 has a Renilla luciferase (Rluc) driven by the 35S promoter that is used as an internal control. A splicing reporter consisting of an intron with flanking exon sequences linked to the firefly luciferase gene was inserted into the expression cassette (Figure 5A). Three different splicing reporter constructs were generated depending on the intron to be tested. One intron-containing construct was from the Waxy gene (intron Wx), one from SR45a, and one from HSFTF9. For the detection of the cis-effect of the candidate motifs for the binding by the RBD to the RNAs, the mutated candidate motifs were generated and inserted into the expression cassette. Co-expression of the splicing reporter construct and empty vector (pAN578) was used as a control (VC, empty vector control). Luciferase activity measurements were carried out according to the manufacturer’s instructions (E1910, Promega, www.promega.com). Four biological replicates were used. RT-PCR was carried out as described above except for 300 ng total RNA were used for the cDNA synthesis.

### Sequence analysis

All the gene IDs mentioned were from maize B73 V4 version (http://plants.ensembl.org/Zea_mays/Info/Index, https://www.maizegdb.org), except some as indicated. All the gene IDs mentioned in the phylogenetic tree were from EnsemblPlants (http://plants.ensembl.org/index.html). Multiple sequence analyses were carried out using ClustalW2 (https://www.ebi.ac.uk/Tools/msa/clustalw2/). Conserved domain analysis was performed using Conserved Domain Search Service (CD Search; https://www.ncbi.nlm.nih.gov/Structure/cdd/wrpsb.cgi). 2D and 3D structure of SR45a was predicted using Phyre2 (http://www.sbg.bio.ic.ac.uk/∼phyre2/html/page.cgi?id=index). 

## Accession numbers

*CALCIUM UNDERACCUMULATION 1* (Zm00001d004109), SRP34 (Zm00001d040672), SR45a (Zm00001d047847, SR45a), MAPKKK3 (Zm00001d026511; Zm00001d025449), NCRK (Zm00001d014967), NF-YB1 (Zm00001d010574), CONSTANS-LIKE9 (Zm00001d045804), HSFTF9 (Zm00001d048041), Phosphatidylethanolamine-binding protein genes (Zm00001d046300, Zm00001d006116, Zm00001d021135), mRNP protein (Zm00001d051139), SCL33 (Zm00001d022243), SR34 (Zm00001d040672), SRP (Zm00001d051139), SPFcc1-like (Zm00001d048415), waxy (Zm00001d045462).

## Footnotes

Data deposition: Raw RNA sequencing data, transcriptional and alternative splicing analysis have been deposited in the NCBI Gene Expression Omnibus (GEO) database (GSE154373 and GSE167670).

## Supplemental data

The following materials are available in the online version of this article.

**Supplemental Figure S1**. Maize plants were grown under different environmental conditions in the Enviratron.

**Supplemental Figure S2**. Venn diagram showing the abundance of the differential alternative splicing (DAS) types.

**Supplemental Figure S3**. GO enrichment analysis for differentially spliced genes.

**Supplemental Figure S4**. Differential alternative splicing in selected regulatory genes.

**Supplemental Figure S5**. Differential alternative splicing in PE-binding protein genes in response to increased MDTs.

**Supplemental Figure S6**. Differential alternative splicing in selected SR genes and *HSFTF9* in response to increased MDTs.

**Supplemental Figure S7**. RT-qPCR validation of the two major group isoforms of *SR45a* and *HSFTF9* in response to increased MDTs.

**Supplemental Figure S8**. Multiple short uORFs in the 5′ UTR of the group 2 *SR45a* genes in maize.

**Supplemental Figure S9**. Alternative splicing analysis of *SR45a* in maize inbred line W22 and multiple short uORFs in the 5′ UTR of the group 2 *SR45a* genes in maize.

**Supplemental Figure S10**. Phylogeny of SR45a protein genes.

**Supplemental Figure S11**. Expression patterns of the *SR45a* genes in Arabidopsis and maize.

**Supplemental Figure S12**. Amino acid sequence alignment of the SR45a isoforms (Zm00001d047847 in maize B73 (Version 4)).

**Supplemental Figure S13**. Alignment between maize and Arabidopsis SR45a protein isoforms.

**Supplemental Figure S14**. RNA splicing assay.

**Supplemental Figure S15**. RNA splicing assay of the *SR45a* isoforms with their upstream regions.

**Supplemental Figure S16**. Predicted 2D structure of Group 1 SR45a (P005).

**Supplemental Figure S17**. Sequence alignment of the CDS and translated protein of *SR45a* from maize B73 and W22.

**Supplemental Figure S18**. Sequence alignment of three *SR45a* isoforms amplified from maize inbred line W22.

**Supplemental Table S1.** Reads and mapping of the sequence samples.

**Supplemental Table S2.** Alternative splicing overview in all the samples.

**Supplemental Table S3.** Overview of the differentially alternative splicing (DAS) events in W22 response to increased MDTs.

**Supplemental Table S4.** Differential alternative splicing events and the corresponding gene expression changes in W22 in response to increased MDTs (20 DAG).

**Supplemental Table S5.** Differential alternative splicing events and the corresponding gene expression changes in W22 in response to increased MDTs (27 DAG).

**Supplemental Table S6.** All the DEGs identified in maize W22 in response to the increased MDTs.

**Supplemental Table S7.** GO enrichment analysis of the identified differential alternative splicing events.

**Supplemental Table S8.** The number of reads mapped to the exon inclusion isoform and the exon skipping isoform of the genes used to generate the heatmap in Figure 2, Figure S4 and Figure S5.

**Supplemental Table S9.** Primers used in this paper.

## Supplementary Material

kiab110_Supplementary_Data

## Notes

Z.L. and S.H.H. designed the research; Z.L. and J.T. performed the research; Z.L., D.C.B., and S.H.H. analyzed the data; D.C.B. and S.H.H. provided oversight, Z.L., D.C.B., and S.H.H. wrote the article.

The author responsible for distribution of materials integral to the findings presented in this article in accordance with the policy described in the Instructions for Authors (https://academic.oup.com/plphys/pages/general-instructions) is: Stephen H. Howell (shh@iastate.edu).
