# Erratum to: Daily temperature cycles promote alternative splicing of RNAs encoding SR45a, a splicing regulator in maize

**DOI:** 10.1093/plphys/kiab163

**Published:** 2021-05-16

**Authors:** Zhaoxia Li, Jie Tang, Diane C Bassham, Stephen H Howell

DOI: https://doi.org/10.1093/plphys/kiab110; *Plant Physiology*, 2021.

In the originally published version of this manuscript, the section title was incorrectly included as part of the article title. The incorrect title was: “Signalling and Response Daily temperature cycles promote alternative splicing of RNAs encoding SR45a, a splicing regulator in maize”.

The correct title is: “Daily temperature cycles promote alternative splicing of RNAs encoding SR45a, a splicing regulator in maize”. This has been corrected online.

