# Supplementary material for: Daily temperature cycles promote alternative splicing of RNAs encoding SR45a, a splicing regulator in maize
Source: Plant Physiol. 2021 Mar 10;186(2):1318–35. doi: 10.1093/plphys/kiab110 (PMC8195531; doi:10.1093/plphys/kiab110)
Supplement: kiab110_Supplementary_Data [file kiab110_supplementary_data.zip › pp.00114.2021-s01.pdf]

Supplement to: Daily temperature cycles promote alternative splicing of RNAs encoding SR45a, a splicing regulator in maize

Zhaoxia Li[a], Jie Tang[b], Diane C. Bassham[b] and Stephen H. Howell[a,1]
[a]Plant Sciences Institute, Iowa State University, Ames, Iowa, 50011
[b]Genetics, Development and Cell Biology Department, Iowa State University, Ames, Iowa, 50011

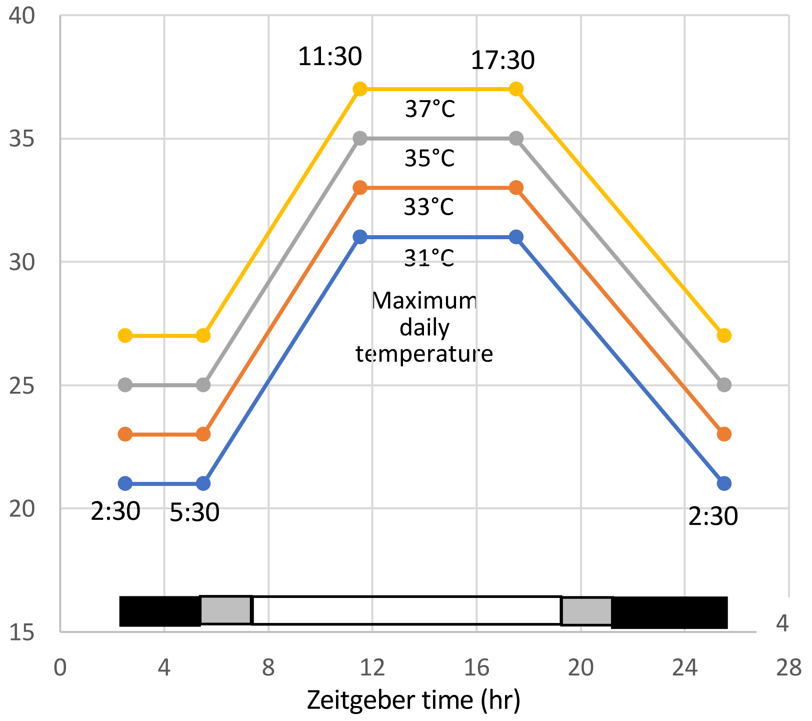

Supplemental Figure S1. Maize plants were grown under different environmental conditions in the Enviratron.
Maize plants (W22) were subjected to photoperiod and temperature cycles in which maximum daily temperatures (MDT) reached 31, 33, 35 or 37°C in the different growth chambers of the Enviratron. Morning and evening temperatures were ramped up or down over 6 and 8 hr, respectively, while light intensities were ramped up or down over 2 hr (grey part of illumination bar). V4 (20 DAG) and V5 (27 DAG) plants were sampled for RNAseq analysis at ZT 11:30. For analysis of daily fluctuations in the abundance of RNA isoforms, plants were sampled every 2 hr in the virtual morning to early afternoon.

Supplemental Figure S1

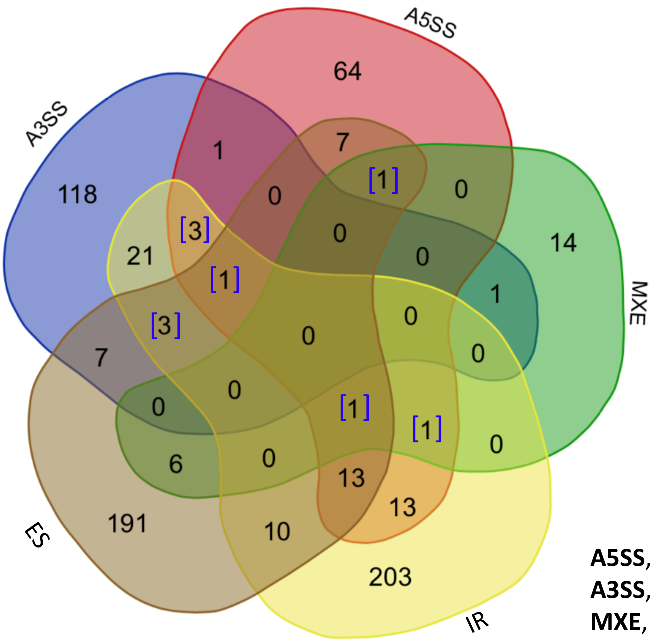

[1] A3SS A5SS IR ES	Zm00001d040672 SRP34
[1] A3SS A5SS IR ES	Zm00001d006116 FT
[1] A5SS MXE ES	Zm00001d047847 SR45a
[1] A5SS MXE IR	Zm00001d004109 CAU1
[3] A3SS IR ES	Zm00001d016777 expressed protein<br>Zm00001d025665 amino acid permease 2<br>Zm00001d041801 RNA-binding KH domain containing protein
[3] A3SS A5SS IR	Zm00001d008933 expressed protein<br>Zm00001d021247 expressed protein<br>Zm00001d038805 YebC-related

A5SS, Alternative 5' Splicing Site;
A3SS, Alternative 3' Splicing Site;
MXE, Mutually exclusive exons;
IR, Retained Intron; **ES**, Exon Skipped

Supplemental Figure S2. Venn diagram showing the abundance of the differential alternative splicing (DAS) types.
89 of the 679 (13.10%) differentially spliced genes at increased MDT have multiple DAS types. 10 of them have more than 3 types of DASs as indicated.

Supplemental Figure S2

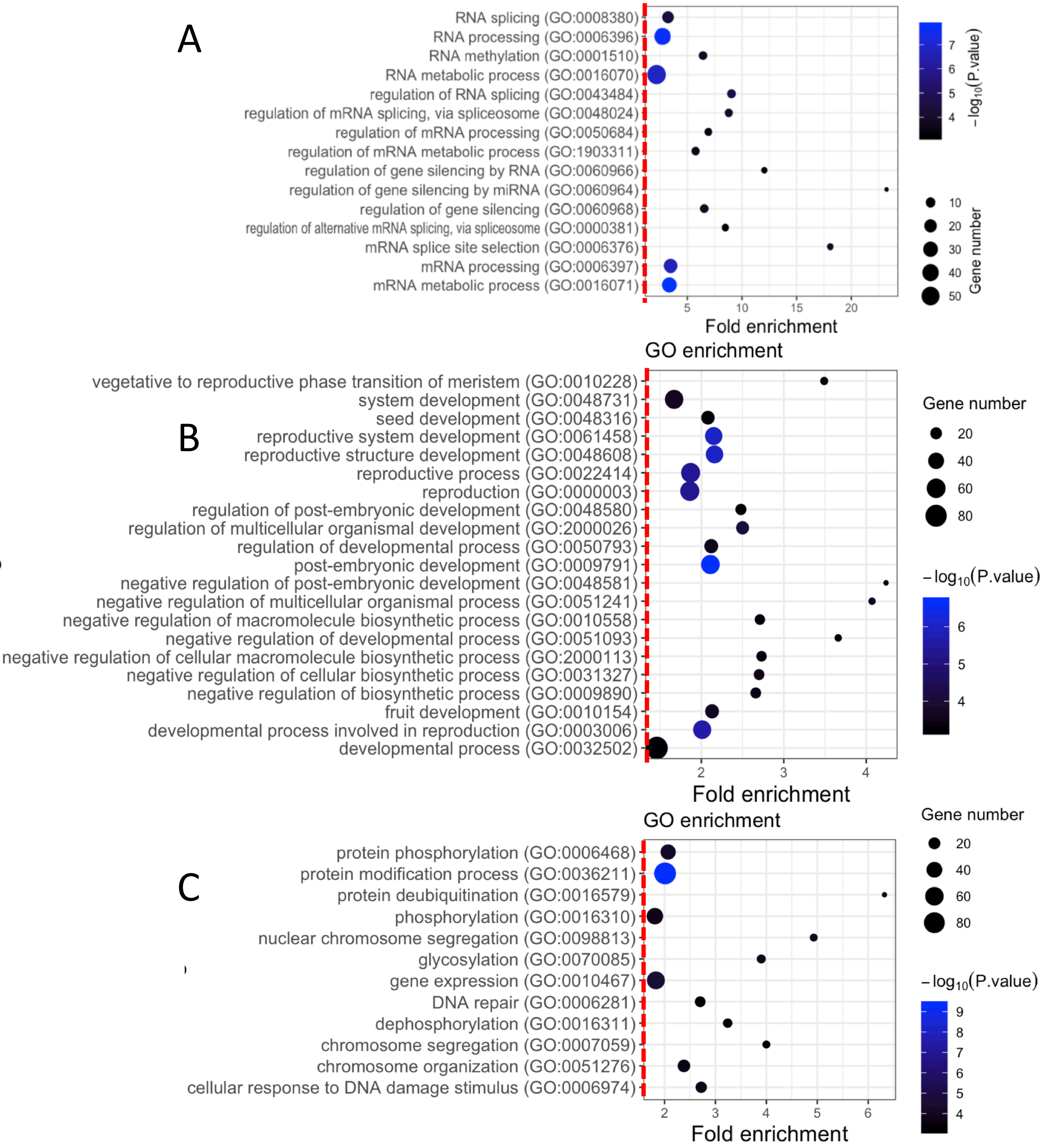

Supplemental Figure S3. GO enrichment analysis for differentially spliced genes. Diagrams show the enrichment of the differential alternative splicing (DAS) genes in response to increased MDTs. GO terms for (A) RNA processing (B) plant development and (C) protein phosphorylation/chromosome segregation. X axis indicates fold enrichment. Color of dot represents P value and size of dot represents the number of genes with the indicated fold enrichment.

Supplemental Figure S3

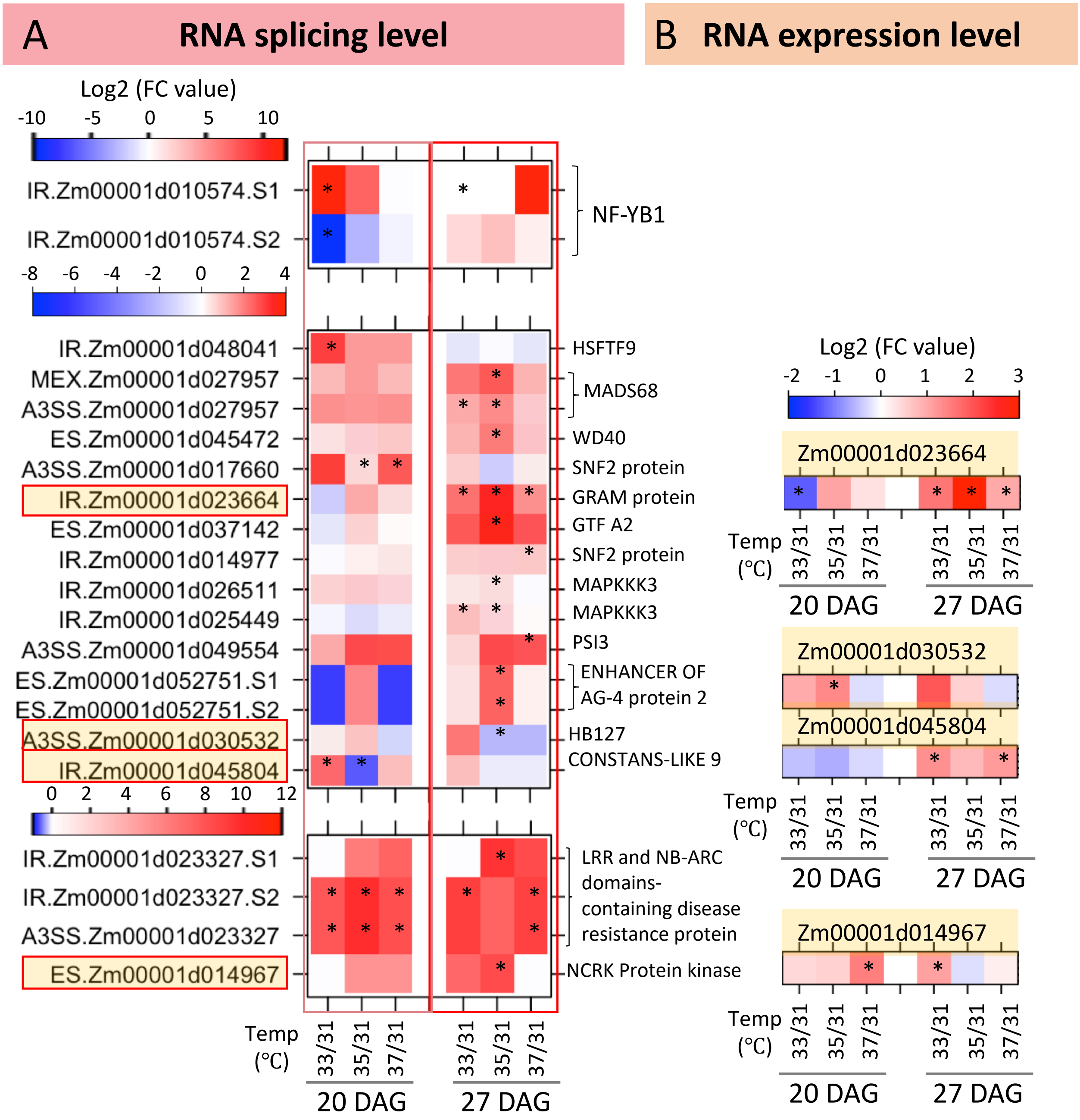

Supplemental Figure S4. Differential alternative splicing in selected regulatory genes. (A) Heat map of differential alternative splicing (DAS) of key regulatory genes in response to increased MDTs. (B) Heat map of changes in expression of these genes in response to increased MDTs. Note two different developmental stages (V4, 20 DAG and V5, 27 DAG) and different MDTs. * indicates significant differences. See the legend in Figure 2 for the explanation of the comparisons of the heatmap.

Supplemental Figure S4

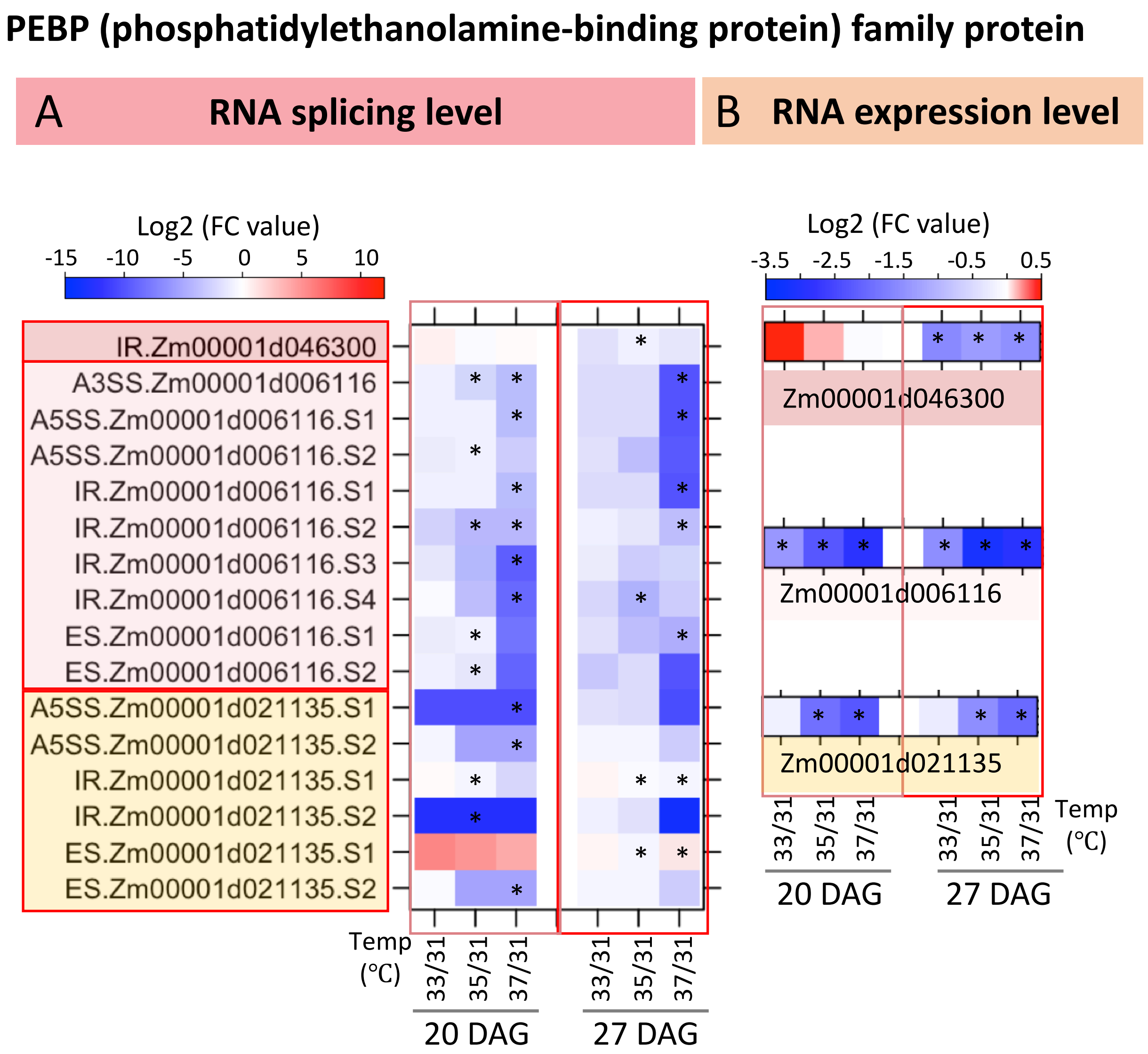

Supplemental Figure S5. Differential alternative splicing in PE-binding protein genes in response to increased MDTs
(A) DAS genes encoding phosphatidylethanolamine-binding proteins in response to increased MDTs. (B) Differential expression of genes shown in panel A that are involved in plant development in response to increased MDTs. Note two different developmental stages (V4, 20 DAG and V5, 27 DAG) and different MDTs. * indicates the significant differences.

Supplemental Figure S5

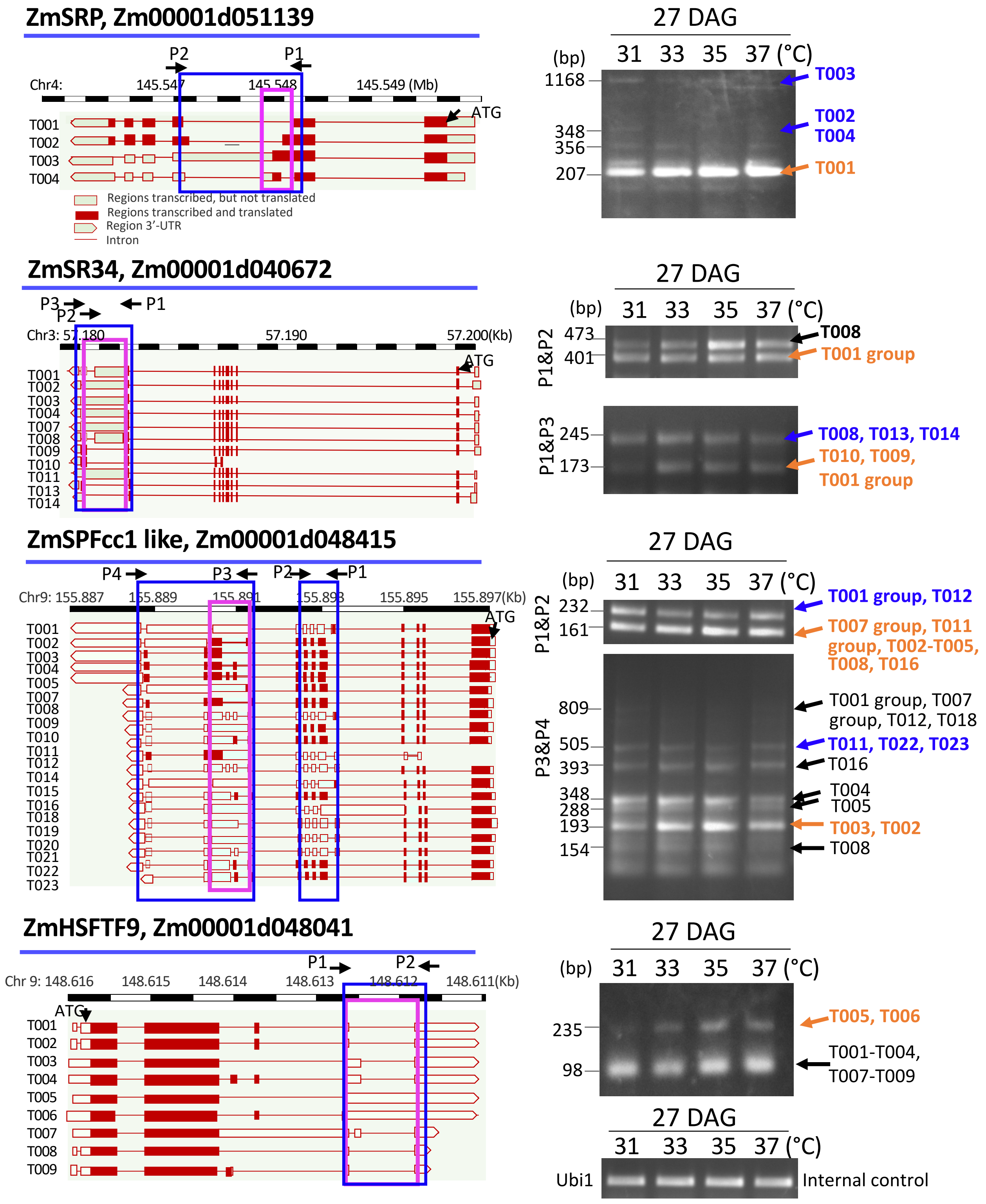

Supplemental Figure S6

Supplemental Figure S6. Differential alternative splicing in selected *SR* genes and *HSFTF9* in response to increased MDTs

Gene models (left, B73 (V4 version)) and RT-PCR (right) show DAS changes in *SR* genes and *HSFTF9* in response to increased MDTs. Box indicates the region in which amplicons were generated and in which heat-induced DASs occur as ascertained from our RNAseq data. Amplicon patterns show changes in SR genes and HSFTF9 RNA isoforms in response to increased MDTs. V4 and V5 plants from chambers with different MDTs were sampled for RNA analysis at ZT 11:30. Maize *Ubi1* was used as the internal control. V5 plants were used for RT-PCR validation of the DAS from RNAseq data. Orange arrows indicate amplicons representing isoforms that increase with increasing MDT, while blue arrows indicate isoforms that decline under these conditions, and black arrows indicate isoforms that comparable to the basal temperature.

Supplemental Figure S6 continued

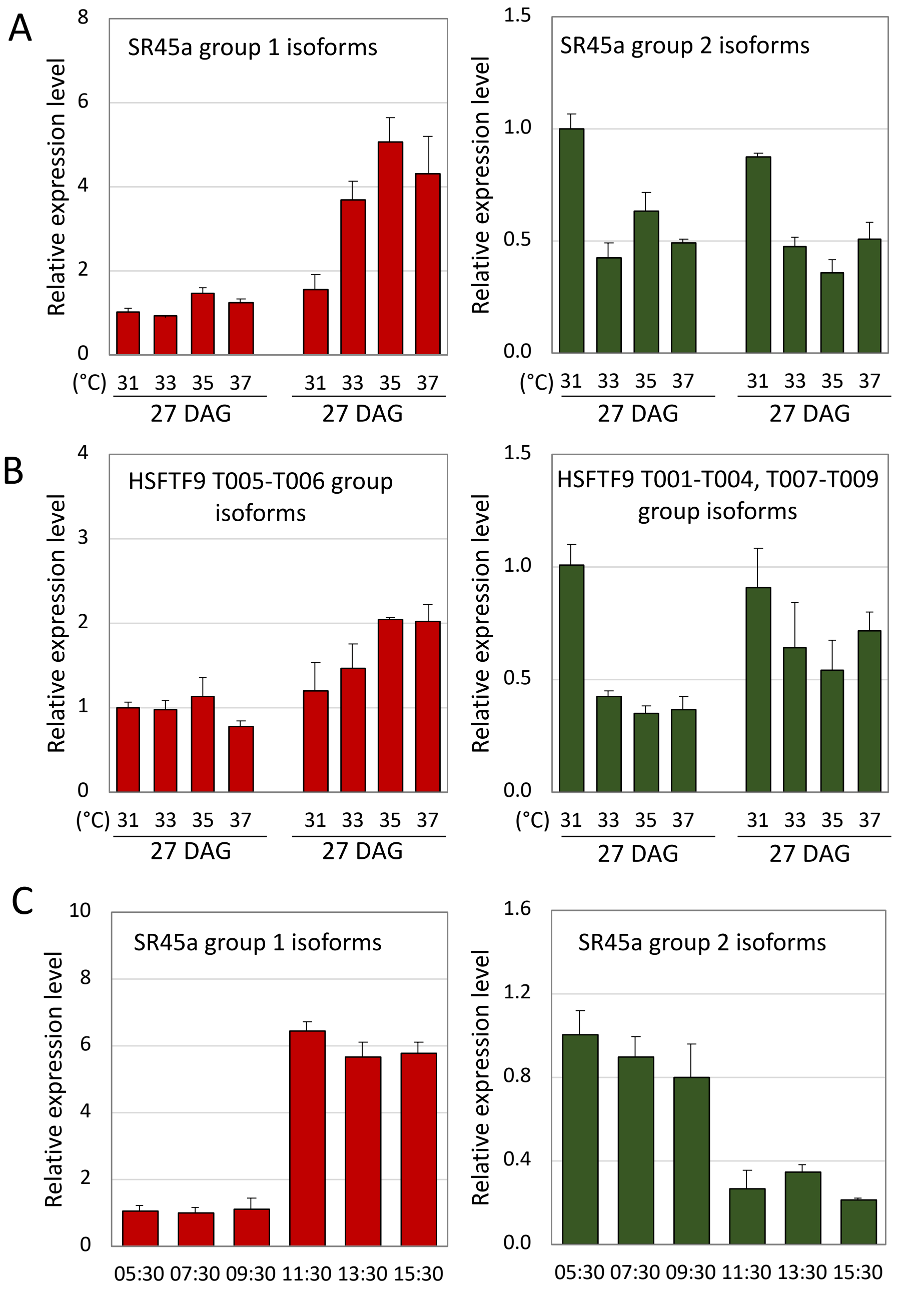

Supplemental Figure S7. RT-qPCR validation of the two major group isoforms of *SR45a* and *HSFTF9* in response to increased MDTs

(A) and (B) RT-qPCR validation of the two major group isoforms of *SR45a* (A) and *HSFTF9* (B) in response to increased MDTs. (C) Time course of daily changes in *SR45a* RNA isoforms in response to different MDTs (37°C, same as Figure 4C) by using RT-qPCR. Values are the means of three replicates ± SD. Expression levels of genes were analyzed by RT-qPCR, fold changes in transcripts were calculated by the 2-ΔΔCt method with maize *Ubi1* as an internal control and the values for normal conditions (31°C, 20 DAG) were set as 1 fold.

Supplemental Figure S7

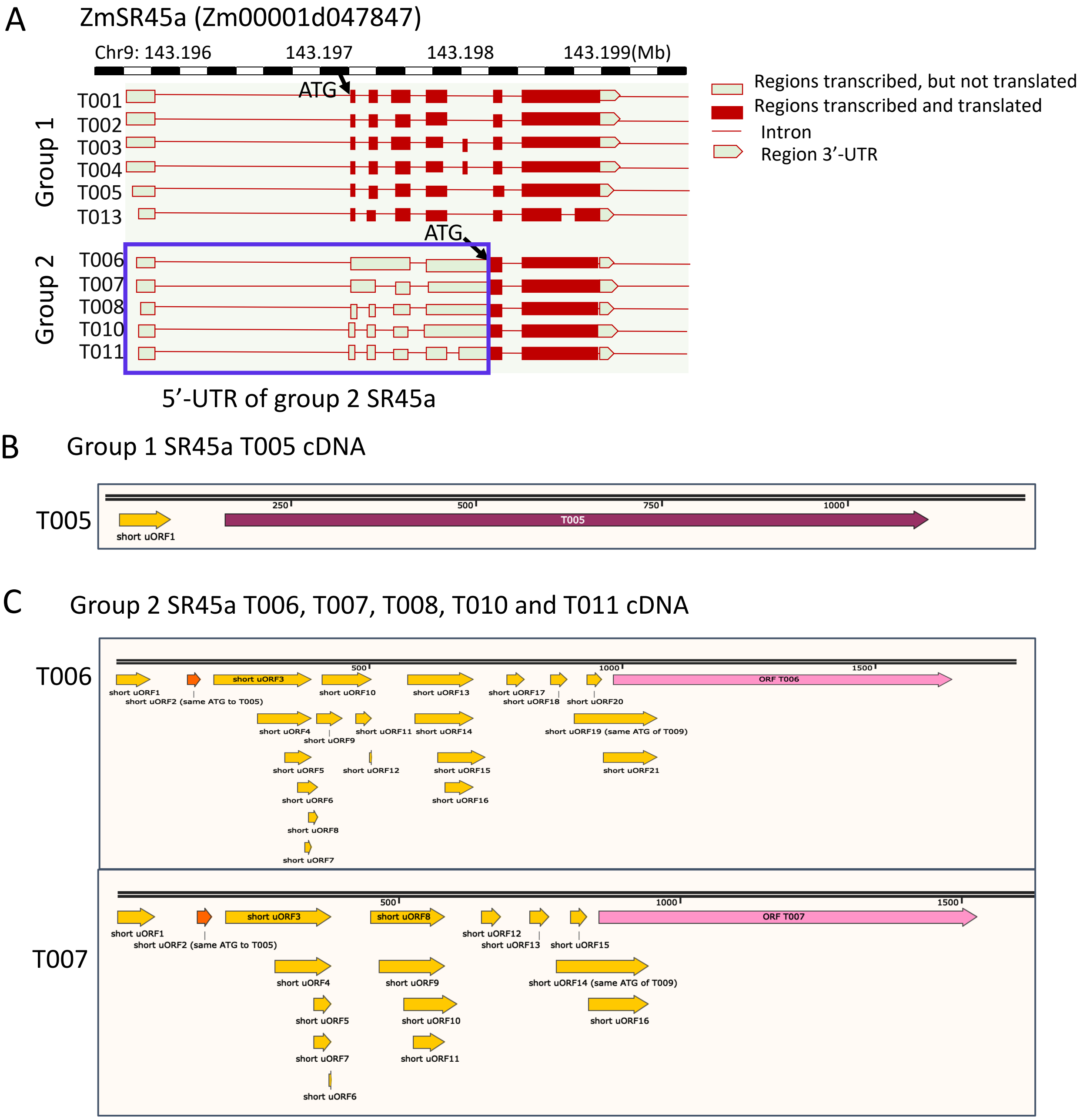

Supplemental Figure S8

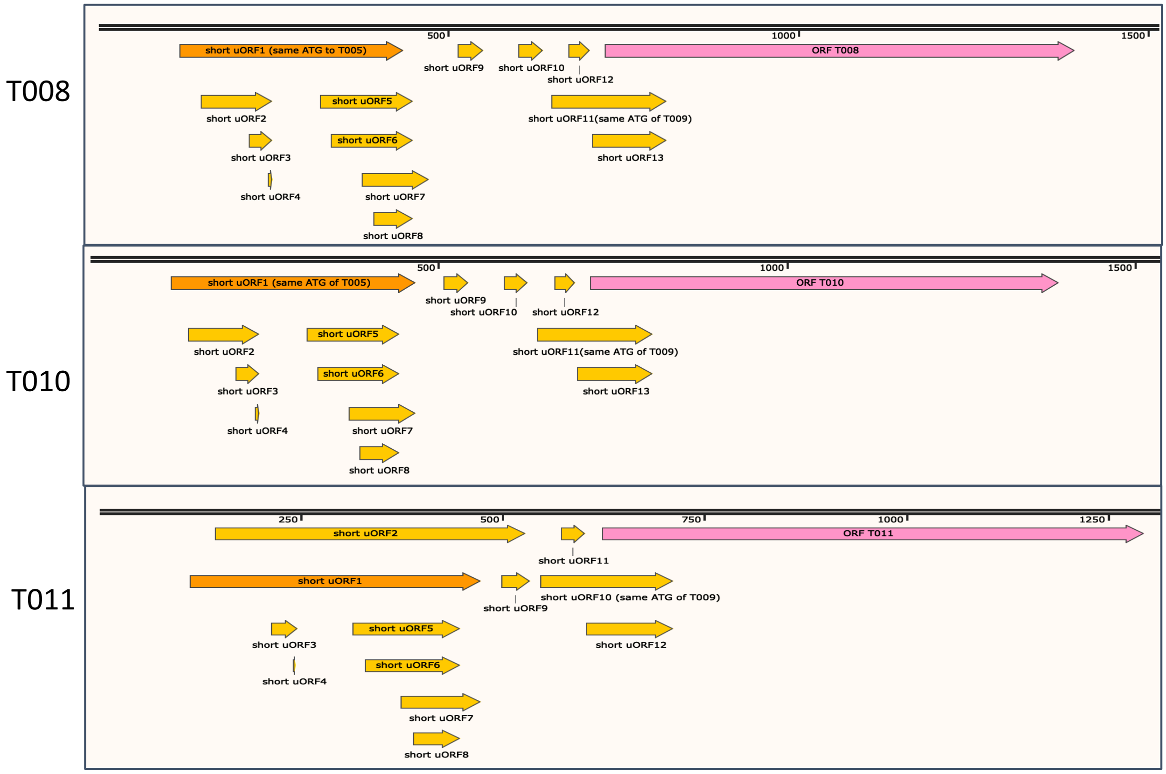

Supplemental Figure S8. Multiple short uORFs in the 5’ UTR of the group 2 *SR45a* genes in maize

(A) Gene models B73 (V4 version) show the diversity of the 5’ UTR of group 2 *SR45a* genes in maize due to the AS. Box indicates the 5’ UTR region based on the longest ORF. (B) and (C) The predicted short uORFs in the 5’ UTR of group2 *SR45a* genes in inbred line B73 but not in the group 1 *SR45a* genes, which is the feature of NMD targets.

Supplemental Figure S8 continued

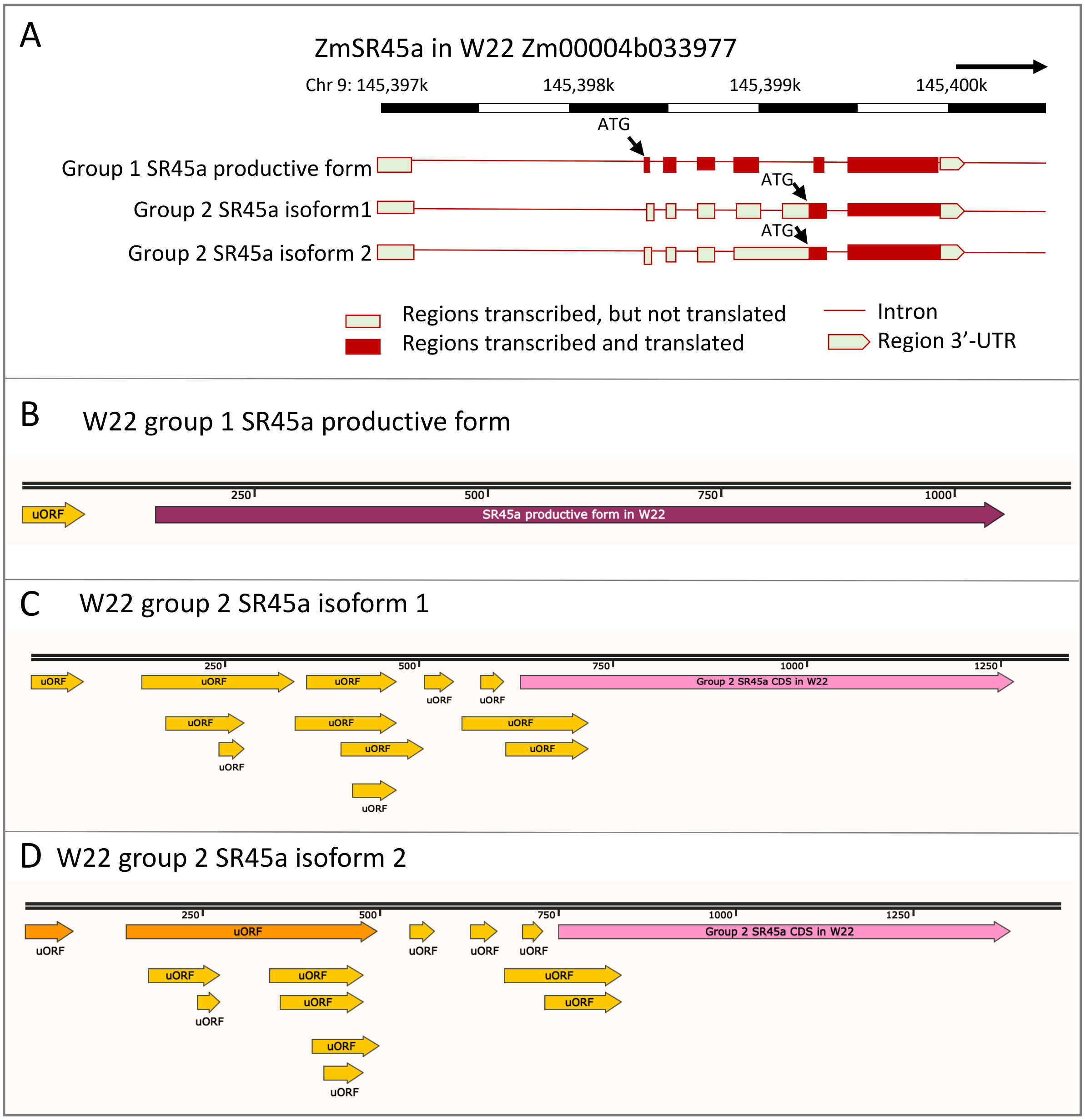

Supplemental Figure S9. Alternative splicing analysis of *AR45a* in maize inbred line W22 and multiple short uORFs in the 5' UTR of the group 2 *SR45a* genes in maize

(A) Alternative splicing analysis of *SR45a* in maize inbred line W22. Three of the *SR45a* RNA isoforms from maize inbred line W22 were amplified and sequenced. The amplified isoforms were alignment with the *SR45a* genome sequence in maize line W22. A group 1 *SR45a* productive from corresponding to T005 in B73 and two group 2 *SR45a* isoforms similar to T007 and T008 (based on the AS pattern and feature of the 5'UTR) were identified in maize W22. (B), (C) and (D) The predicted short uORFs in the 5' UTR of group2 *SR45a* genes in inbred line W22 but not in the group 1 *SR45a* genes, which is similar to that in Maize line B73 (Supplemental Figure 7).

Supplemental Figure S9

A

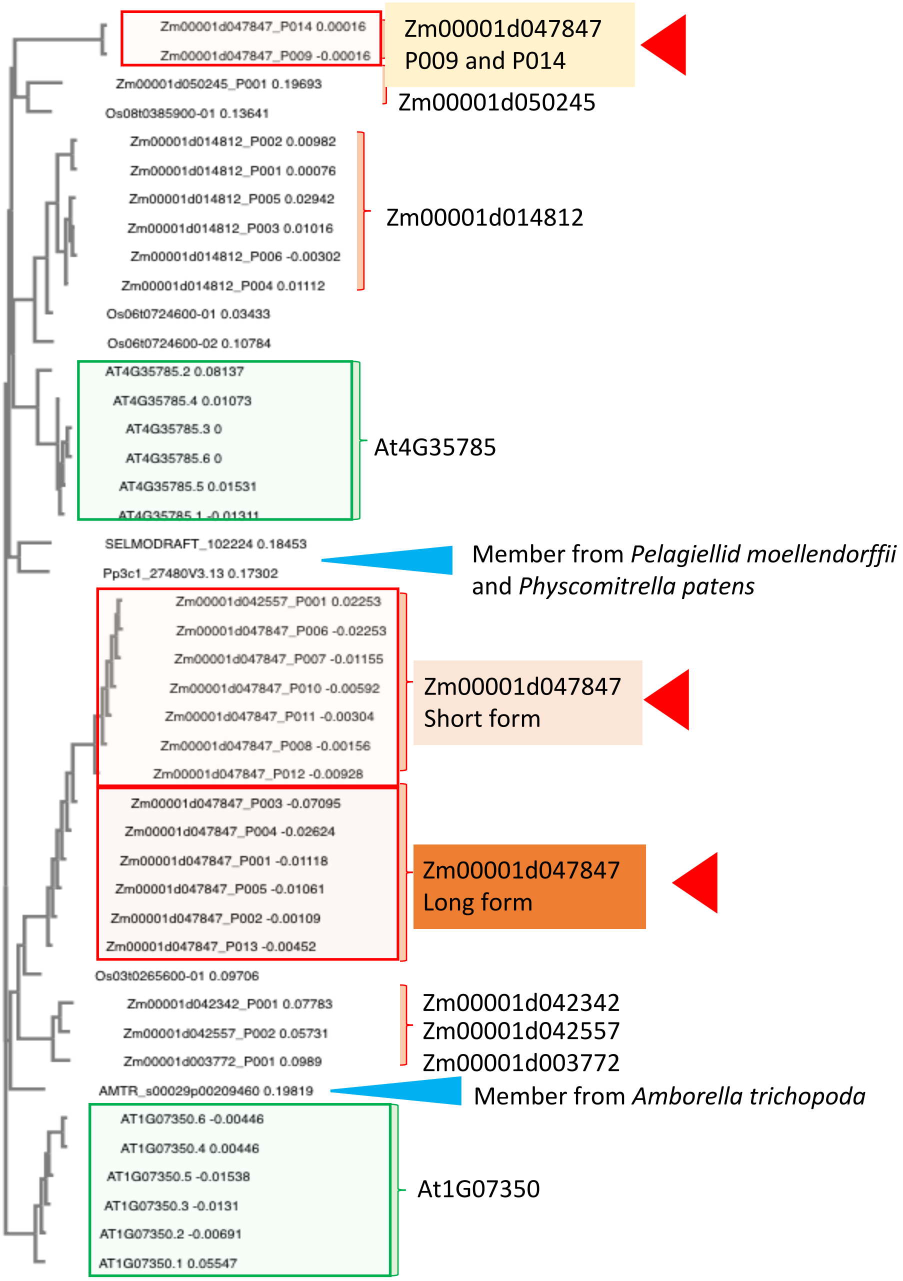

B

Synteny relationship of SR45a genes in Arabidopsis, rice and maize					
Arabidopsis thaliana genes	Location	Oryza sativa homologues	Location	Zea mays homologues	Location
AT4G35785	4:16952963-16955342	Os06g0724600	6:30779796-30784595	Zm00001d014812	5:63969221-63973693
SR45A (AT1G07350)	1:2257384-2260187	Os03g0265600	3:8767643-8770548	Zm00001d042557	3:171678085-171684082
				Zm00001d042342	3:162156155-162162138
				Zm00001d003772	2:58357086-58360109
No homologues		Os08g0385900	8:18189406-18195703	Zm00001d050245	4:75823246-75830658
No homologues		No homologues		Zm00001d047847	9:143195591-143199191

Supplemental Figure S10

Supplemental Figure S10. Phylogeny of SR45a protein genes

(A) Amino acid sequences of the SR45a genes in Arabidopsis, rice, maize and other species. In this clade there is one SR45a gene in Arabidopsis, three genes in rice and six in maize. The focus of our study, ZmSR45a (Zm00001d047847, red triangle labeled) is one of the six genes in maize.

(B) Syntenic relationship among the Arabidopsis, rice and maize genes in the SR45a clade. Three maize genes (Zm00001d042342, Zm00001d042577 and Zm00001d003772) are syntenic to AtSR45a, and all three are expressed at a level too low to detect and might be pseudogenes. For the gene featured in this study, Zm00001d047847, no homologs in orthologous genome regions were found in Arabidopsis and rice. Data summarized based on Ensembl Plants Synteny analysis in Comparative Genomics.

Supplemental Figure S10 continued

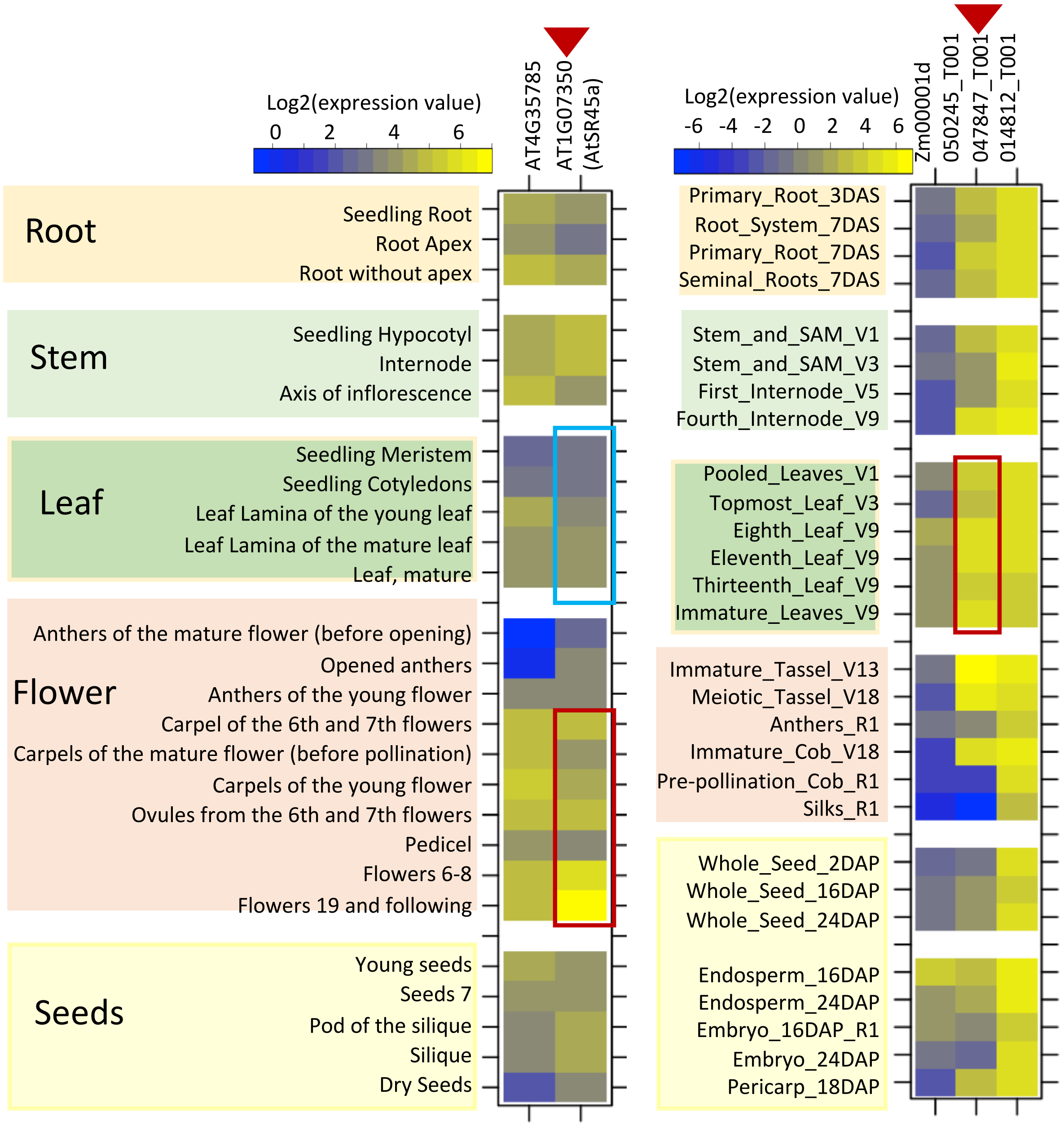

Supplemental Figure S11. Expression patterns of the *SR45a* genes in Arabidopsis and maize. *ZmSR45a* (Zm00001d047847, red triangle) is highly expressed in leaves, tassels and ears. *AtSR45a* was highly expressed in flowers, but not so much in leaves. Expression data from gene models in TAIR (www.arabidopsis.org) and maize GDB (www.maizegdb.org). Heat maps were generated using log2 of the expression values.

Supplemental Figure S11

```
                                                Phosphorylation sites
Zm00001d047847_P013     MSYSRGS----SERYGSRSPYSRSTRYRSRSRSVDSSDAENPGNNLYVTGLSARVTDRDL 56
Zm00001d047847_P005     MSYSRGS----SERYGSRSPYSRSTRYRSRSRSVDSSDAENPGNNLYVTGLSARVTDRDL 56
Zm00001d047847_P001     MSYSRGSSFFTSERYGSRSPYSRSTRYRSRSRSVDSSDAENPGNNLYVTGLSARVTDRDL 60
Zm00001d047847_P002     MSY------SRGSRYGSRSPYSRSTRYRSRSRSVDSSDAENPGNNLYVTGLSARVTDRDL 54
Zm00001d047847_P006     ------------------------------------------------------------ 0
Zm00001d047847_P012     ------------------------------------------------------------ 0
                                 N- RS rich region                  RRM_SF super
                                                                    family (RBD)

                                                        RNA recognition motif (RRM) domain
Zm00001d047847_P013     EKHFSTEGEVIDASVVYDPWTRESRGFGFVTMAAVKDADRCIKYLDRSVLQGRVITVEKA 116
Zm00001d047847_P005     EKHFSTEGEVIDASVVYDPWTRESRGFGFVTMAAVKDADRCIKYLDRSVLQGRVITVEKA 116
Zm00001d047847_P001     EKHFSTEGEVIDASVVYDPWTRESRGFGFVTMAAVKDADRCIKYLDRSVLQGRVITVEKA 120
Zm00001d047847_P002     EKHFSTEGEVIDASVVYDPWTRESRGFGFVTMAAVKDADRCIKYLDRSVLQGRVITVEKA 114
Zm00001d047847_P006     ---------------------------------MIALLQSCFWYQRHFLIW---SPITQA 24
Zm00001d047847_P012     ----------------------------------------------------MPTSAKGA 8
                                                                                   *
                                   RRM_SF super family (RBD)
                                   RNA recognition motif (RRM) domain

Zm00001d047847_P013     KRRRGRTPTPGKYLGTKSSRGRRYSTSYSPDRRGRYSSRYSPDRRDCHGRDRSYSPYDRR 176
Zm00001d047847_P005     KRRRGRTPTPGKYLGTKSSRGRRYSTSYSPDRRGRYSSRYSPDRRDCHGRDRSYSPYDRR 176
Zm00001d047847_P001     KRRRGRTPTPGKYLGTKSSRGRRYSTSYSPDRRGRYSSRYSPDRRDCHGRDRSYSPYDRR 180
Zm00001d047847_P002     KRRRGRTPTPGKYLGTKSSRGRRYSTSYSPDRRGRYSSRYSPDRRDCHGRDRSYSPYDRR 174
Zm00001d047847_P006     KRRRGRTPTPGKYLGTKSSRGRRYSTSYSPDRRGRYSSRYSPDRRDCHGRDRSYSPYDRR 84
Zm00001d047847_P012     KRRRGRTPTPGKYLGTKSSRGRRYSTSYSPDRRGRYSSRYSPDRRDCHGRDRSYSPYDRR 68
                        ************************************************************
                                   RNA recognition motif (RRM) domain

Zm00001d047847_P013     RSYSPYGRDESYSPYYGRRSYSPYYSRRYRSRSPYCYRRRRSPSVSPYYRRH-------- 228
Zm00001d047847_P005     RSYSPYGRDESYSPYYGRRSYSPYYSRRYRSRSPYCYRRRRSPSVSPYYRRHRYRSVSKS 236
Zm00001d047847_P001     RSYSPYGRDESYSPYYGRRSYSPYYSRRYRSRSPYCYRRRRSPSVSPYYRRHRYRSVSKS 240
Zm00001d047847_P002     RSYSPYGRDESYSPYYGRRSYSPYYSRRYRSRSPYCYRRRRSPSVSPYYRRHRYRSVSKS 234
Zm00001d047847_P006     RSYSPYGRDESYSPYYGRRSYSPYYSRRYRSRSPYCYRRRRSPSVSPYYRRHRYRSVSKS 144
Zm00001d047847_P012     RSYSPYGRDESYSPYYGRRSYSPYYSRRYRSRSPYCYRRRRSPSVSPYYRRHRYRSVSKS 128
                        ****************************************************
                              RNA recognition motif (RRM) domain

Zm00001d047847_P013     ------------------------SHSPESERSTSYSPKKGSKREPSRSRYSGKSRHSRE 264
Zm00001d047847_P005     SSSSPRARGRSYSCSLSPQRSYSRSHSPESERSTSYSPKKGSKREPSRSRYSGKSRHSRE 296
Zm00001d047847_P001     SSSSPRARGRSYSCSLSPQRSYSRSHSPESERSTSYSPKKGSKREPSRSRYSGKSRHSRE 300
Zm00001d047847_P002     SSSSPRARGRSYSCSLSPQRSYSRSHSPESERSTSYSPKKGSKREPSRSRYSGKSRHSRE 294
Zm00001d047847_P006     SSSSPRARGRSYSCSLSPQRSYSRSHSPESERSTSYSPKKGSKREPSRSRYSGKSRHSRE 204
Zm00001d047847_P012     SSSSPRARGRSYSCSLSPQRSYSRSHSPESERSTSYSPKKGSKREPSRSRYSGKSRHSRE 188
                                                ************************************
                                          C- RS rich region

Zm00001d047847_P013     SHSHSRSSYSRSVSRERSA     283
Zm00001d047847_P005     SHSHSRSSYSRSVSRERSA     315
Zm00001d047847_P001     SHSHSRSSYSRSVSRERSA     319
Zm00001d047847_P002     SHSHSRSSYSRSVSRERSA     313
Zm00001d047847_P006     SHSHSRSSYSRSVSRERSA     223
Zm00001d047847_P012     SHSHSRSSYSRSVSRERSA     207
                        *******************
                          C- RS rich region
```

Supplemental Figure S12. Amino acid sequence alignment of the SR45a isoforms (Zm00001d047847 in maize B73 (Version 4)).
Yellow highlight indicates serine (S) residues and purple indicates arginine (R). Other domains in the protein are as indicated.

Supplemental Figure S12

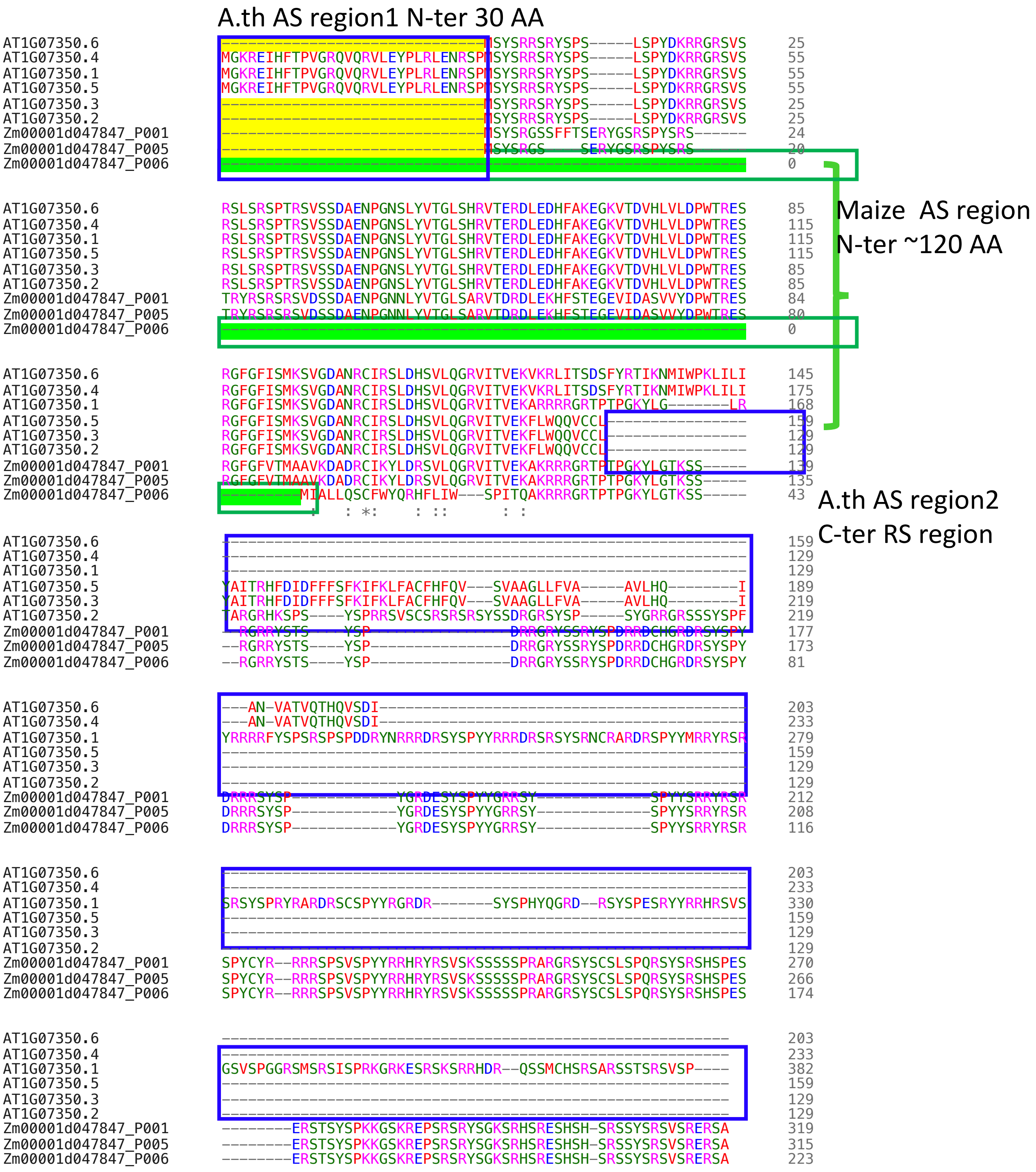

Supplemental Figure S13. Alignment between maize and Arabidopsis SR45a isoforms. The blue boxes represent two AS regions in Arabidopsis reported by Tanabe et al., 2007. One region missing in the shorter Arabidopsis isoforms contains the 30 N-terminal amino acids, the other region is composed of the C-terminal RS region. Arabidopsis sequences are compared to Group 1 maize SR45a (P001, P005) and Group 2 (P006) sequences. The N-terminal 117 amino acids in the maize SR45a corresponding to the RS domain and RBD, which is missing in the shorter forms, is outlined with a green box.

Supplemental Figure S13

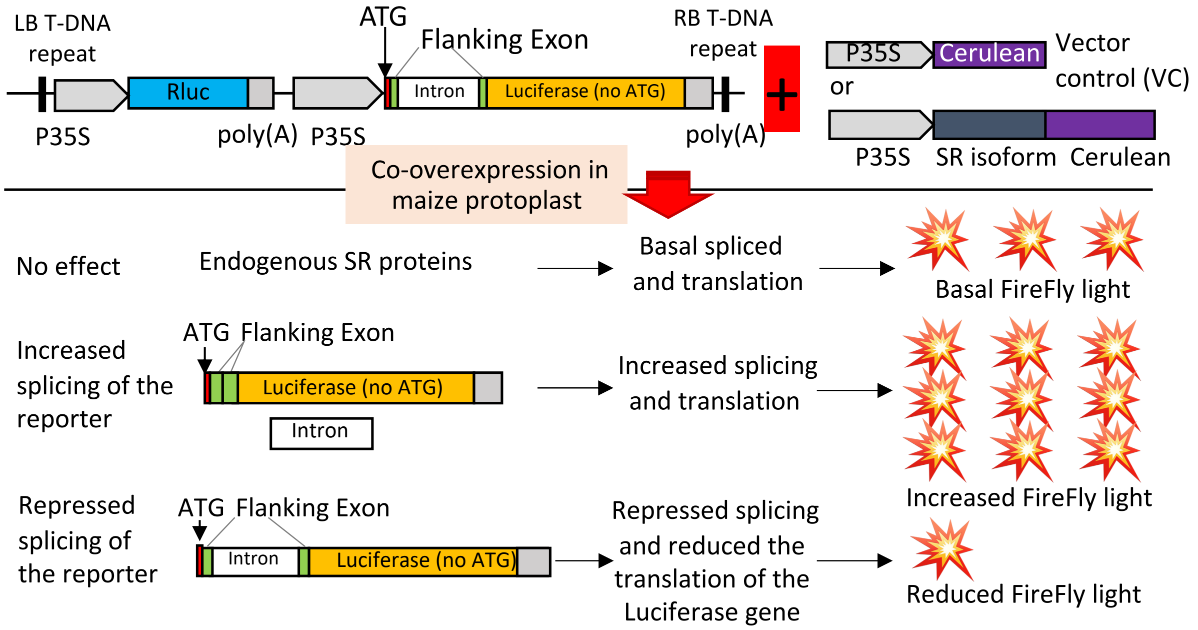

Supplemental Figure S14. RNA splicing assay.

As in Figure 5A, a dual-luciferase reporter was used to assess RNA splicing efficiency in a maize protoplast transient expression system. The reporter consists of an intron, linked to the firefly luciferase gene, and splicing out of the intron promotes expression of the firefly luciferase reporter. Different recombinant forms of SR45a co-transfected with the luciferase reporter were tested for RNA splicing efficiency. In the empty vector control, a basal level of splicing activity is expected from the action of endogenous splicing factors. However, expression of the *SR45a* isoforms are expected to increase or repress splicing of the intron–containing substrates.

Supplemental Figure S14

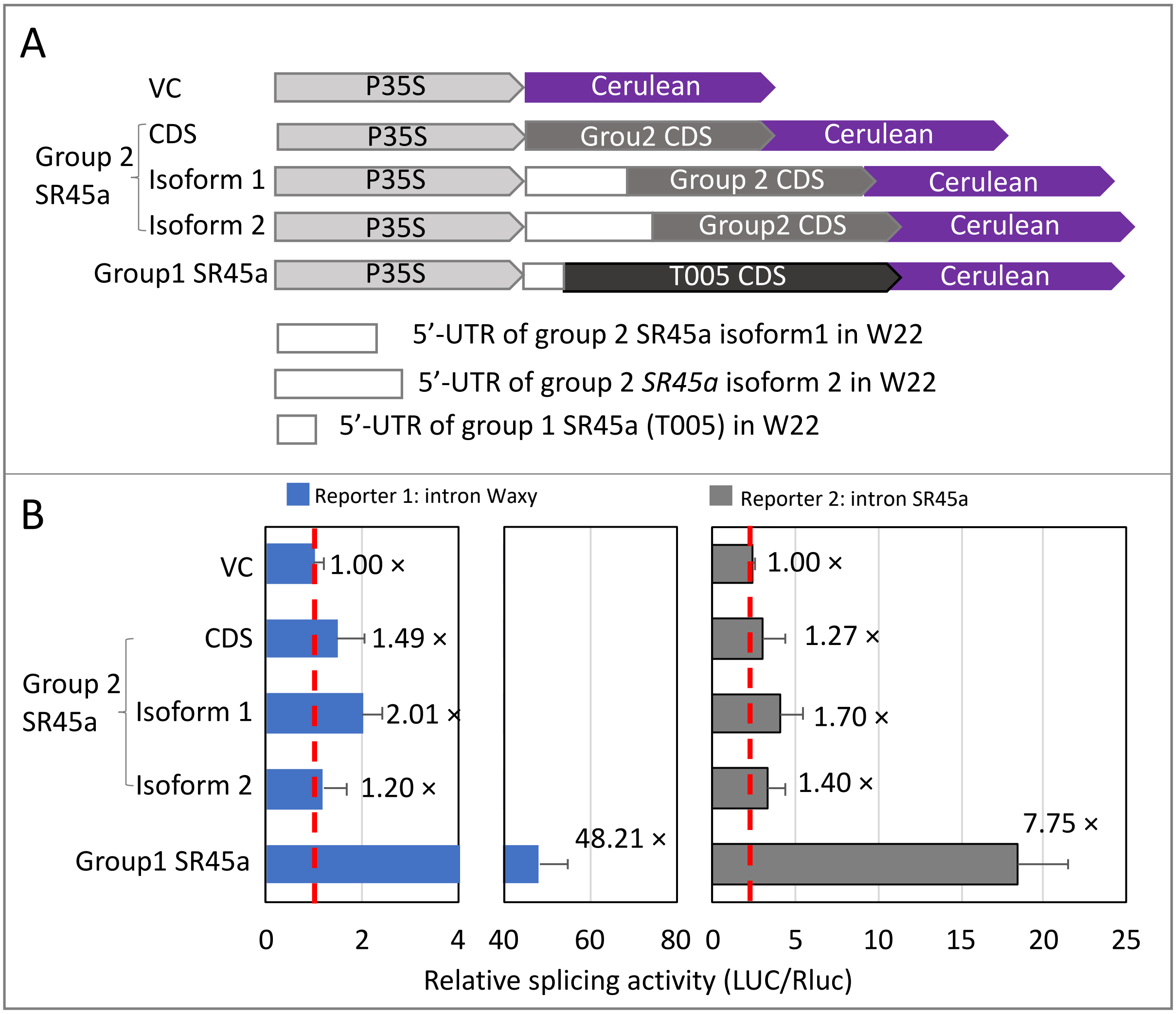

Supplemental Figure S15. RNA splicing assay of the SR45a isoforms with their upstream regions.

(A) SR45a isoforms for RNA splicing assay. As in Figure 5A and Figure 13, a dual-luciferase reporter was used to assess RNA splicing efficiency in a maize protoplast transient expression system. Different recombinant forms of SR45a co-transfected with the luciferase reporter were tested for RNA splicing efficiency. Group 2 SR45a CDS was the coding region of group 2 SR45a in maize inbred line W22. Group 2 SR45a isoforms 1 and 2 were the two group 2 SR45a isoforms amplified from maize inbred line W22, they were predicted with the same group 2 SR45a CDS, however, with different 5′-UTR produced by AS. The different 5′-UTR had different features as shown in Supplemental Figure 7D. (B) Splicing efficiency of the different SR45a protein isoforms with or without the upstream region. Splicing efficiency activity is expressed as the relative activity of firefly luciferase versus Renilla luciferase. Values are the means of the four replicates ± SD. Values indicate the fold increase in firefly luciferase activity resulting from the expression of the SR45a isoforms. Empty pAN578 (the backbone of SR gene overexpression constructs) was used as a control (VC, 1-fold).

Supplemental Figure S15

2-D structure of ZmSR45a and the difference between ZmSR45a P005 and P006

Th N-terminal region of SR45a has several predicted alpha-helical and beta-sheet structures, within the RS domain and the RNA binding domain (RBD). The RBD is **90 amino acids** in length and consists of a **four-stranded beta-sheet packed against two alpha-helices**. The N-terminal arginine/serine rich domain (RS domain) has multiple predicted phosphorylation sites. This region is absent in Group 2 isoforms, such as in P006.

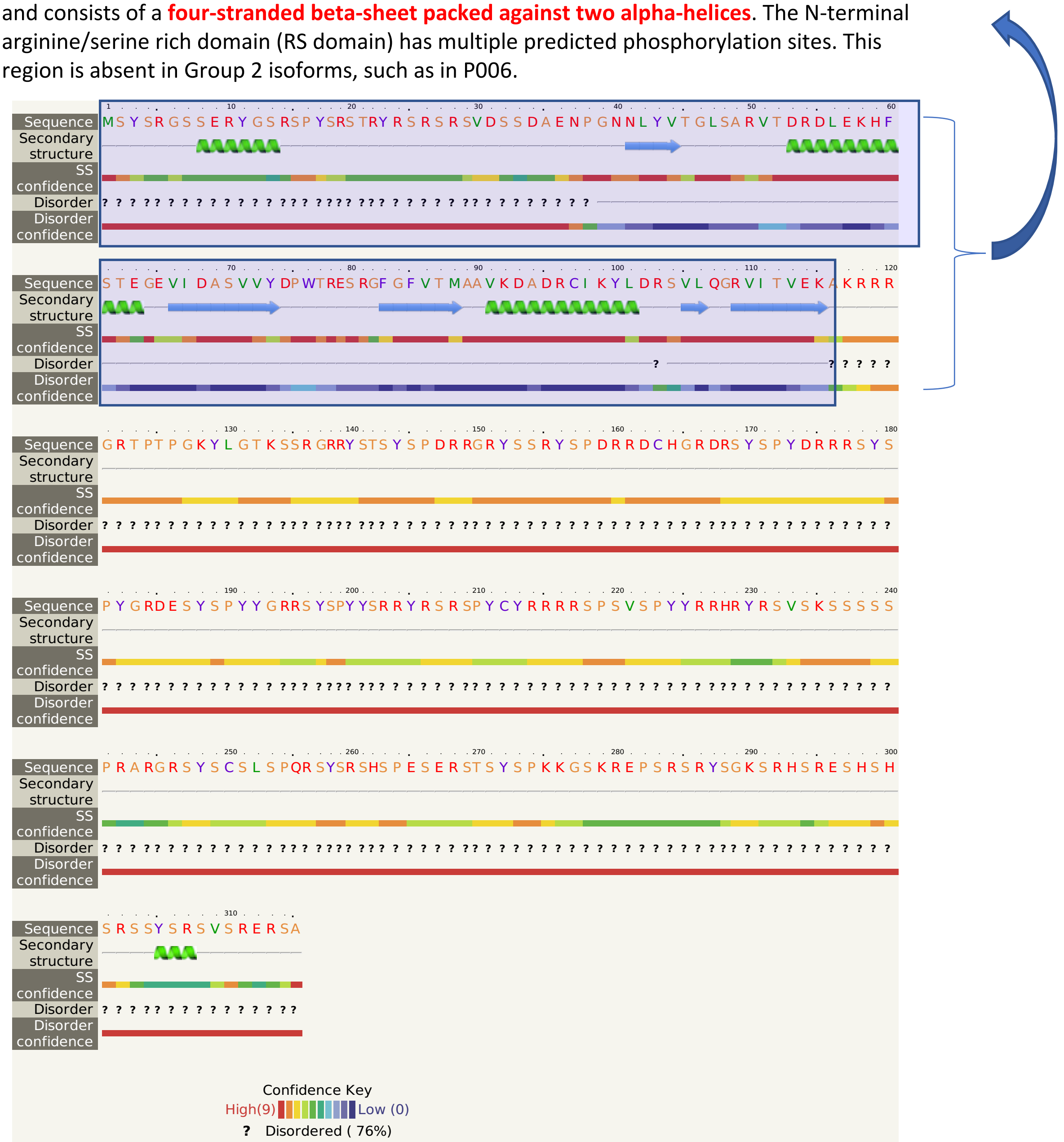

Supplemental Figure S16. Predicted 2D structure of a Group 1 SR45a (P005). 2D structure was predicted using Phyre2 (http://www.sbg.bio.ic.ac.uk/~phyre2/html/page.cgi?id=index). The N-terminal ~120 aa (containing the RBD) missing in Group 2 isoforms (as in P006) are boxed in. Note that most of the C-terminal region is intrinsically disordered.

Supplemental Figure S16

```
B73 (V4) SR45a T005  1    ATGTCTTACTCAAGGGGCTCAAGTGAAAGGTATGGATCTCGCTCACCTTACTCGAGAAGC  60
                          |||||||||||||||||||||||||||||||||||||||||||  |||||||||||||||
W22 SR45a T005       1    ATGTCTTACTCAAGGGGCTCAAGTGAAAGGTATGGATCTCGCTTGCCTTACTCGAGAAGC  60

B73 (V4) SR45a T005  61   ACAAGGTACCGAAGTCGCTCTAGGAGTGTCGACTCTAGTGATGCTGAAAACCCTGGGAAC  120
                          ||||||||||||||||||||||||||||||||||||||||||||||||||||||||||||
W22 SR45a T005       61   ACAAGGTACCGAAGTCGCTCTAGGAGTGTCGACTCTAGTGATGCTGAAAACCCTGGGAAC  120

B73 (V4) SR45a T005  121  AACCTGTATGTGACTGGTTTGTCGGCTCGTGTAACGGATCGAGATCTGGAGAAGCACTTC  180
                          ||||||||||||||||||||||| ||||||||||||||||||||||||||||||||||||
W22 SR45a T005       121  AACCTGTATGTGACTGGTTTGTCAGCTCGTGTAACGGATCGAGATCTGGAGAAGCACTTC  180

B73 (V4) SR45a T005  181  TCCACTGAGGGTGAGGTGATTGATGCAAGCGTTGTGTATGATCCTTGGACAAGGGAATCA  240
                          |||||| |||||||||||||||||||||||||||| ||||||||||||||||||||||||
W22 SR45a T005       181  TCCACTAAGGGTGAGGTGATTGATGCAAGCGTTGTATATGATCCTTGGACAAGGGAATCA  240

B73 (V4) SR45a T005  241  CGGGGGTTTGGTTTTGTTACCATGGCTGCTGTTAAAGATGCAGATCGCTGCATCAAATAT  300
                          ||||| ||||||||||||||||||||||||||||||||||||||||||||||||||||||
W22 SR45a T005       241  CGGGGTTTTGGTTTTGTTACCATGGCTGCTGTTAAAGATGCAGATCGCTGCATCAAATAT  300

B73 (V4) SR45a T005  301  CTGGACCGTTCTGTGTTGCAAGGTCGGGTCATAACTGTTGAGAAGGCAAAGAGAAGACGA  360
                          ||||||||||||||||||||||||||||||||||||||||||||||||||||||||||||
W22 SR45a T005       301  CTGGACCGTTCTGTGTTGCAAGGTCGGGTCATAACTGTTGAGAAGGCAAAGAGAAGACGA  360

B73 (V4) SR45a T005  361  GGTAGAACCCCAACACCTGGGAAGTATCTTGGCACAAAATCATCCCGTGGACGGAGGTAC  420
                          ||||||||||||||||||||||||||||||||||||||||||||||||||||||||||||
W22 SR45a T005       361  GGTAGAACCCCAACACCTGGGAAGTATCTTGGCACAAAATCATCCCGTGGACGGAGGTAC  420

B73 (V4) SR45a T005  421  TCCACAAGCTACTCACCTGATCGGAGGGGCCGTTACAGTTCCCGCTACTCACCTGATCGG  480
                          |||||||||||                                    |||||||||||||
W22 SR45a T005       421  TCCACAAGCTA------------------------------------CTCACCTGATCGG  444

B73 (V4) SR45a T005  481  AGGGACTGTCATGGTAGAGACCGGTCATACTCTCCATATGACAGAAGGCGATCATACTCT  540
                          |||| | |||||||||||||||||||||||||||||||||||||||||||||||||||||
W22 SR45a T005       445  AGGGGCCGTCATGGTAGAGACCGGTCATACTCTCCATATGACAGAAGGCGATCATACTCT  504

B73 (V4) SR45a T005  541  CCTTACGGCAGGGATGAATCATATTCCCCTTACTATGGCCGGCGATCATACTCACCTTAC  600
                          || ||| ||||||||| |||||||||||||||||||||||||||||||||||||||||||
W22 SR45a T005       505  CCCTACAGCAGGGATGGATCATATTCCCCTTACTATGGCCGGCGATCATACTCACCTTAC  564

B73 (V4) SR45a T005  601  TACAGCAGACGGTATCGTTCAAGGTCCCCATACTGCTACAGAAGACGCAGGTCACCTTCT  660
                          || |||||||||||||||||||||||||||||||||||||||||||| ||||||||||||
W22 SR45a T005       565  TATAGCAGACGGTATCGTTCAAGGTCCCCATACTGCTACAGAAGACGTAGGTCACCTTCT  624

B73 (V4) SR45a T005  661  GTTTCGCCATACTACCGCCGGCACAGGTACAGATCTGTCTCCAAGTCATCCAGTTCATCT  720
                          |||||||||||||||||||||||||||||||||||||| |||||||||||||||||||||
W22 SR45a T005       625  GTTTCGCCATACTACCGCCGGCACAGGTACAGATCTGTGTCCAAGTCATCCAGTTCATCT  684

B73 (V4) SR45a T005  721  CCAAGGGCCAGAGGACGGAGCTATTCGTGCAGTTTATCACCACAGAGAAGCTACTCGCGC  780
                          ||||||||||||||||||||||||||||||||||||||||||||||||||||||||||||
W22 SR45a T005       685  CCAAGGGCCAGAGGACGGAGCTATTCGTGCAGTTTATCACCACAGAGAAGCTACTCGCGC  744

B73 (V4) SR45a T005  781  AGCCATTCCCCAGAATCCGAGAGATCAACAAGCTATTCTCCTAAGAAAGGGAGTAAAAGG  840
                          ||||||||||||||||||||||||||||||||||||||||||||||||||||||| ||||
W22 SR45a T005       745  AGCCATTCCCCAGAATCCGAGAGATCAACAAGCTATTCTCCTAAGAAAGGGAGTAGAAGG  804

B73 (V4) SR45a T005  841  GAACCCTCGCGTAGCAGATACTCTGGCAAGAGTCGTCATTCAAGGGAAAGCCACTCTCAT  900
                          ||| ||||||||||||||||||||||||||||||||||||||||||||||| ||||||||
W22 SR45a T005       805  GAATCCTCGCGTAGCAGATACTCTGGCAAGAGTCGTCATTCAAGGGAAAGCTACTCTCAT  864

B73 (V4) SR45a T005  901  AGCCGCAGTTCGTACTCCAGGTCTGTGTCCAGGGAGCGCTCAGCT  945
                          |||||||||||||||||||||||||||||||||||||||||||||
W22 SR45a T005       865  AGCCGCAGTTCGTACTCCAGGTCTGTGTCCAGGGAGCGCTCAGCT  909


B73 (V4) SR45a P005  1    MSYSRGSSERYGSRSPYSRSTRYRSRSRSVDSSDAENPGNNLYVTGLSARVTDRDLEKHF  60
                          MSYSRGSSERYGSR PYSRSTRYRSRSRSVDSSDAENPGNNLYVTGLSARVTDRDLEKHF
W22 SR45a P005       1    MSYSRGSSERYGSRLPYSRSTRYRSRSRSVDSSDAENPGNNLYVTGLSARVTDRDLEKHF  60

B73 (V4) SR45a P005  61   STEGEVIDASVVYDPWTRESRGFGFVTMAAVKDADRCIKYLDRSVLQGRVITVEKAKRRR  120
                          ST+GEVIDASVVYDPWTRESRGFGFVTMAAVKDADRCIKYLDRSVLQGRVITVEKAKRRR
W22 SR45a P005       61   STKGEVIDASVVYDPWTRESRGFGFVTMAAVKDADRCIKYLDRSVLQGRVITVEKAKRRR  120

B73 (V4) SR45a P005  121  GRTPTPGKYLGTKSSRGRRYSTSYSPDRRGRYSSRYSPDRRDCHGRDRSYSPYDRRRSYS  180
                          GRTPTPGKYLGTKSSRGRRYSTSYSPDRRGR+               RSYSPYDRRRSYS
W22 SR45a P005       121  GRTPTPGKYLGTKSSRGRRYSTSYSPDRRGRHGRD------------RSYSPYDRRRSYS  168

B73 (V4) SR45a P005  181  PYGRDESYSPYYGRRSYSPYYSRRYRSRSPYCYRRRRSPSVSPYYRRHRYRSVSKSSSSS  240
                          PY RD SYSPYYGRRSYSPYYSRRYRSRSPYCYRRRRSPSVSPYYRRHRYRSVSKSSSSS
W22 SR45a P005       169  PYSRDGSYSPYYGRRSYSPYYSRRYRSRSPYCYRRRRSPSVSPYYRRHRYRSVSKSSSSS  228

B73 (V4) SR45a P005  241  PRARGRSYSCSLSPQRSYSRSHSPESERSTSYSPKKGSKREPSRSRYSGKSRH  293
                          PRARGRSYSCSLSPQRSYSRSHSPESERSTSYSPKKGS+RE SRSRYSGKSRH
W22 SR45a P005       229  PRARGRSYSCSLSPQRSYSRSHSPESERSTSYSPKKGSRRESSRSRYSGKSRH  281
```

Supplemental Figure S17. Sequence alignment of the CDS and translated protein of SR45a from maize B73 and W22.

Supplemental Figure S17

>SR45a productive form in W22

ATGCACAGGCGGACTGGCGGACTCGGGGCTCGTACTAACAGGAAAAAAGGCCCAGAGGCAGTGCCGTGACCGATTCGAGACGCAAGAGAAGG
ATCGAGACGAGGCGGCGGTTTTCCGGGGGCAGCATCGTCTCGGCCACGAAGATGTCTTACTCAAGAGGCTCAAGTGAAAGGTATGGATCTCGCT
TGCCTTACTCGAGAAGCACAAGGTACCGAAGTCGCTCTAGGAGTGTCGACTCTAGTGATGCTGAAAACCCTGGGAACAACCTGTATGTGACTGG
TTTGTCAGCTCGTGTAACGGATCGAGATCTGGAGAAGCACTTCTCCACTAAGGGTGAGGTGATTGATGCAAGCGTTGTATATGATCCTTGGACAA
GGGAATCACGGGGTTTTGGTTTTGTTACCATGGCTGCTGTTAAAGATGCAGATCGCTGCATCAAATATCTGGACCGTTCTGTGTTGCAAGGTCGG
GTCATAACTGTTGAGAAGGCAAAGAGAAGACGAGGTAGAACCCCAACACCTGGGAAGTATCTTGGCACAAAATCATCCCGTGGACGGAGGTAC
TCCACAAGCTACTCACCTGATCGGAGGGGCCGTCATGGTAGAGACCGGTCATACTCTCCATATGACAGAAGGCGATCATACTCTCCCTACAGCAG
GGATGGATCATATTCCCCTTACTATGGCCGGCGATCATACTCACCTTACTATAGCAGACGGTATCGTTCAAGGTCCCCATACTGCTACAGAAGAC
GTAGGTCACCTTCTGTTTCGCCATACTACCGCCGGCACAGGTACAGATCTGTGTCCAAGTCATCCAGTTCATCTCCAAGGGCCAGAGGACGGAGC
TATTCGTGCAGTTTATCACCACAGAGAAGCTACTCGCGCAGCCATTCCCCAGAATCCGAGAGATCAACAAGCTATTCTCCTAAGAAAGGGAGTAG
AAGGGAATCCTCGCGTAGCAGATACTCTGGCAAGAGTCGTCATTCAAGGGAAAGCTACTCTCATAGCCGCAGTTCGTACTCCAGGTCTGTGTCTA
GGGAGCGCTCAGCCTGATCTGCGAGGTGTGTCGTCCTAGGTGGTTATAAGATATGCGCTGTATTTGCATCATTCAGAACTAATC

>Goup2 SR45a isoform 1 in W22 (Seq3-29) upstream 631bp

ATGCACAGGCGGACTGGCGGACTCGGGGCTCGTACTAACAGGAAAAAAGGCCCAGAGGCAGTGCCGTGACCGATTCGAGACGCAAGAGAAGG
ATCGAGACGAGGCGGCGGTTTTCCGGGGGCAGCATCGTCTCGGCCACGAAGATGTCTTACTCAAGAGGCTCAAGTGAAAGGTATGGATCTCGCT
TGCCTTACTCGAGAAGCACAAGGTACCGAAGTCGCTCTAGGAGTGTCGACTCTAGTGATGCTGAAAACCCTGGGAACAACCTGTATGTGACTGG
TTTGTCAGCTCGTGTAACGGATCGAGATCTGGAGAAGCACTTCTCCACTAAGGGTGATTGATGCAAGCGTTGTATATGATCCTTGGACAAGGGA
ATCACGGGGTTTTGGTTTTGTTACCATGGCTGCTGTTAAAGATGCAGATCGCTGCATCAAATATCTGGACCGTTCTGTGTTGCAAGGTCGGGTCA
TAACTGTTGAGAAGGTTTTGTTGACAGCAACAGGGTAGATGTTGGTAGCTGCTCTGCAACAAACTGATACAACCTAACACCAAACCATGCCAACC
TCAGCCAAAGGGTTGTATGCCCTACATTGTATATTATTTTTCTTAAATATGTTTATTCAGATTGTAGATGATTGCTCTCTTGCAGTCTTGCTTTTGGT
ATCAAAGACACTTCCTTATTTGGTCCTCTATTACACAGGCAAAGAGAAGACGAGGTAGAACCCCAACACCTGGGAAGTATCTTGGCACAAAATCA
TCCCGTGGACGGAGGTACTCCACAAGCTACTCACCTGATCGGAGGGGCCGTCATGGTAGAGACCGGTCATACTCTCCATATGACAGAAGGCGAT
CATACTCTCCCTACAGCAGGGATGGATCATATTCCCCTTACTATGGCCGGCGATCATACTCACCTTACTATAGCAGACGGTATCGTTCAAGGTCCC
CATACTGCTACAGAAGACGTAGGTCACCTTCTGTTTCGCCATACTACCGCCGGCACAGGTACAGATCTGTGTCCAAGTCATCCAGTTCATCTCCAA
GGGCCAGAGGACGGAGCTATTCGTGCAGTTTATCACCACAGAGAAGCTACTCGCGCAGCCATTCCCCAGAATCCGAGAGATCAACAAGCTATTC
TCCTAAGAAAGGGAGTAGAAGGGAATCCTCGCGTAGCAGATACTCTGGCAAGAGTCGTCATTCAAGGGAAAGCTACTCTCATAGCCGCAGTTCG
TACTCCAGGTCTGTGTCTAGGGAGCGCTCAGCCTGATCTGCGAGGTGTGTCGTCCTAGGTGGTTATAAGATATGCGCTGTATTTGCATCATTCAG
AACTAATC

> Goup2 SR45a isoform 1 in W22 (Seq7-11) upstream 751bp

ATGCACAGGCGGACTGGCGGACTCGGGGCTCGTACTAACAGGAAAAAAGGCCCAGAGGCAGTGCCGTGACCGATTCGAGACGCAAGAGAAGG
ATCGAGACGAGGCGGCGGTTTTCCGGGGGCAGCATCGTCTCGGCCACGAAGATGTCTTACTCAAGAGGCTCAAGTGAAAGGTATGGATCTCGCT
TGCCTTACTCGAGAAGCACAAGGTACCGAAGTCGCTCTAGGAGTGTCGACTCTAGTGATGCTGAAAACCCTGGGAACAACCTGTATGTGACTGG
TTTGTCAGCTCGTGTAACGGATCGAGATCTGGAGAAGCACTTCTCCACTAAGGGTGAGGTAATTGATGCAAGCGTTGTATATGATCCTTGGACAA
GGGAATCACGGGGTTTTGGTTTTGTTACCATGGCTGCTGTTAAAGATGCAGATCGCTGCATCAAATATCTGGACCGTTCTGTGTTGCAAGGTCGG
GTCATAACTGTTGAGAAGGTAAAATCATAAGATTGGGAGCATCTAACTTTCTTATTTAACTTGGCTACTGAATGATATTTATATCATTCCTTAGTAC
TCTACACTGAACATTTTAGTGTTCTGTCCATTGAAGATTTTGTTGACAGCAACAGGGTAGATGTTGGTAGCTGCTCTGCAACAAACTGATACAACC
TAACACCAAACCATGCCAACCTCAGCCAAAGGGTTGTATGCCCTACATTGTATATTATTTTTCTTAAATATGTTTATTCAGATTGTAGATGATTGCT
CTCTTGCAGTCTTGCTTTTGGTATCAAAGACACTTCCTTATTTGGTCCTCTATTACACAGGCAAAGAGAAGACGAGGTAGAACCCCAACACCTGGG
AAGTATCTTGGCACAAAATCATCCCGTGGACGGAGGTACTCCACAAGCTACTCACCTGATCGGAGGGGCCGTCATGGTAGAGACCGGTCATACT
CTCCATATGACAGAAGGCGATCATACTCTCCCTACAGCAGGGATGGATCATATTCCCCTTACTATGGCCGGCGATCATACTCACCTTACTATAGCA
GACGGTATCGTTCAAGGTCCCCATACTGCTACAGAAGACGTAGGTCACCTTCTGTTTCGCCATACTACCGCCGGCACAGGTACAGATCTGTGTCC
AAGTCATCCAGTTCATCTCCAAGGGCCAGAGGACGGAGCTATTCGTGCAGTTTATCACCACAGAGAAGCTACTCGCGCAGCCATTCCCCAGAATC
CGAGAGATCAACAAGCTATTCTCCTAAGAAAGGGAGTAGAAGGGAATCCTCGCGTAGCAGATACTCTGGCAAGAGTCGTCATTCAAGGGAAAG
CTACTCTCATAGCCGCAGTTCGTACTCCAGGTCTGTGTCTAGGGAGCGCTCAGCCTGATCTGCGAGGTGTGTCGTCCTAGGTGGTTATAAGATAT
GCGCTGTATTTGCATCATTCAGAACTAATC

Supplemental Figure S18. Sequence alignment of three SR45a isoforms amplified from maize inbred line W22.
Predicted CDS regions are highlighted in green.

Supplemental Figure S18
